# Supplementary material for: DiffDomain enables identification of structurally reorganized topologically associating domains
Source: Nat Commun. 2024 Jan 13;15:502. doi: 10.1038/s41467-024-44782-6 (PMC10787792; doi:10.1038/s41467-024-44782-6)
Supplement: Supplementary file 1 — Supplementary Information [file 41467_2024_44782_MOESM1_ESM.pdf]

Supplementary Information

DiffDomain enables identification of structurally reorganized topologically associating domains

Dunming Hua[1,2], Ming Gu[1,2], Xiao Zhang[1,2], Yanyi Du[1,2], Hangcheng Xie[1,2], Li Qi[3], Xiangjun Du[1,2], Zhidong Bai[4], Xiaopeng Zhu[5,6], Dechao Tian[1,2,*]

[1]Department of Biostatistics and Systems Biology, School of Public Health (Shenzhen), Sun Yat-sen University, Shenzhen, Guangdong 510275, China. [2]Department of Biostatistics and Systems Biology, School of Public Health (Shenzhen), Shenzhen Campus of Sun Yat-sen University, Shenzhen, 518107, China. [3]Chongqing Municipal Center for Disease Control and Prevention, Chongqing 400042, China. [4]KLASMOE & School of Mathematics and Statistics, Northeast Normal University, Changchun, Jilin 130024, China. [5]MyCellome LLC., Allison Park, PA 15101, USA. [6]Computational Biology Department, School of Computer Science, Carnegie Mellon University, Pittsburgh, PA 15213, USA.

These authors contributed equally: Dunming Hua, Ming Gu, Xiao Zhang, Yanyi Du

Corresponding author: tiandch@mail.sysu.edu.cn

This PDF file includes:

Supplementary Methods 1-7
Supplementary Notes 1-9
Supplementary Tables 1-5
Supplementary Figures 1-30
Supplementary References

Supplementary Methods

Supplementary Method 1 TAD calling for bulk Hi-C data

One of the input of DiffDomain is the TAD list. By default, DiffDomain uses TADs called by Arrowhead [7], which can detect hierarchical TADs and are recommended by Zufferey et al. [18]. Arrowhead with a window size of 2000, resolution of 10 kb, and other parameters as the default is used to call TADs within Hi-C replicates from GM12878 [7]. For other cell lines in [7] that are used in this study, their TAD lists are downloaded from GEO database (GSE63525 [https://www.ncbi.nlm.nih.gov/geo/query/acc.cgi?acc=GSE635 In the case of NHA, DIPG007, and DIPGXIII cell lines, TADs are called by Arrowhead with a window size of 2000, resolution of 10 kb, and other parameters as the default. However, for the mock-infected A549-ACE2 and SARS-CoV-2 infected A549-ACE2 Hi-C data [10], Arrowhead is not used to call TADs. Instead, insulation score method is used for a better comparison with the original study's findings [10]. More specifically, TADs are called using the command 'run-insulator-score-caller.sh', with a binsize of 10 kb, window size of 200 kb, and cutoff of 0.5 (https://github.com/4dn-dcic/docker-4dn-insulator-score-caller), following standard 4D Nucleome consortium protocol and utilizing the same parameters as the original study [10].

Supplementary Method 2 Pseudocode of DiffDomain

The pseudocode of DiffDomain is presented in Supplementary Algorithm 1.

Supplementary Algorithm 1 Hypothesis-testing based method for identifying reorganized TADs.

Require: Contact matrices $\boldsymbol{A}_1$ and $\boldsymbol{A}_2$ of a TAD with N bins in two biological conditions
  $\boldsymbol{D} \leftarrow$ NORMALIZED_DIFFERENCE($\boldsymbol{A}_1, \boldsymbol{A}_2$)
  $\lambda_N \leftarrow$ the largest eigenvalue of $\boldsymbol{D}$
  $\theta_N \leftarrow N^{2/3}(\lambda_N - 2)$
  P value $\leftarrow P_{TW_1}(\theta_N \geq x)$
Ensure: P value

  function NORMALIZED_DIFFERENCE($\boldsymbol{A}_1, \boldsymbol{A}_2$)
    $\boldsymbol{D} \leftarrow \log(\boldsymbol{A}_1) - \log(\boldsymbol{A}_2)$
    for $d = 2 - N, \ldots, N - 2$ **do** ▷ Iterate over d-off diagonal of $\boldsymbol{D}$
      $D_d = (D_{mn}), m - n = d$
      $\mu_d \leftarrow$ mean(D_d)
      $\sigma_d \leftarrow$ standard deviation(D_d)
      $D_{ij} \leftarrow (D_{ij} - \mu_d)/\sigma_d, i - j = d$
    end for
    $\boldsymbol{D} \leftarrow \boldsymbol{D}/\sqrt{N}$
    return $\boldsymbol{D}$
  end function

Supplementary Method 3 Classification of reorganized TADs into different subtypes

The inputs for this step are the subset of condition 1 TADs that are reorganized in condition 2, and the set of condition 2 TADs, which are identified using the same method as that used for calling the condition 1 TADs (Supplementary Method 1). TADs are hierarchically organized, as called by methods such as Arrowhead. Large TADs can be further divided into multiple smaller TADs. In other words, a genomic region may be assigned to multiple TADs. The hierarchical nature of TADs complicates the classification

of reorganized TADs. Before explaining the classification of reorganized TADs, we introduce three essential definitions on pairs of TAD boundaries and pairs of TADs: shared TAD boundaries, identical TADs, and overlapping TADs.

1. Shared TAD boundaries: Two TAD boundaries, one from each condition, are called shared if they are within a given distance. By default, the distance is the maximum value of 30 kb and 10% of the length of the TAD in condition 1. This approach accounts for variations in the length of large TADs, particularly those exceeding 300 kb.
2. Identical TADs: Two TADs, one from condition 1 and the other from condition 2, are called identical if both their left and right boundaries are shared with their corresponding counterparts.
3. Overlapping TADs: A TAD in condition 1 is called overlapping with a TAD in condition 2 if their genomic regions intersect with each other, and they share at most one boundary. Note that identical TADs are not special cases of overlapping TADs based on their definitions.

These three definitions enable us to define relationships among TADs, facilitating the classification of reorganized TADs. Specifically, we use combinations of identical TADs and overlapping TADs to distinguish distinct subtypes of reorganized TADs. The detailed definitions of the subtypes of reorganized TADs are presented below.

1. *Strength-change* TADs: A reorganized TAD is called a *strength-change* TAD if the reorganized TAD in condition 1 has a one-to-one identical relationship with a TAD in condition 2. Specifically, (1) condition 2 has exactly one TAD that is identical to the reorganized TAD, and (2) this condition 2 TAD is not identical to other TADs in condition 1. Note that a reorganized TAD in condition 1 may be identical to multiple TADs in condition 2, or vice versa, due to the broad definition of shared TAD boundaries, which allows for two boundaries to be within a given distance (as defined in the previous paragraph). In such cases, the reorganized TADs are defined as *split* TADs or *merge* TADs (see definitions below). The definition does not take into account the potential TADs in conditions 1 and 2 that overlap with the reorganized TAD, given the hierarchical nature of TADs.
2. *Loss* TADs: A reorganized TAD is called a *loss* TAD if condition 2 has no TAD that overlaps with or is identical to the reorganized TAD.
3. *Split* TADs: A reorganized TAD is called a *split* TAD if the reorganized TAD has either a one-to-many identical relationship or a one-to-many overlapping relationship with TADs in condition 2. In other words, it satisfies one of the following two criteria. First, the reorganized TAD is identical to at least two TADs in condition 2, and none of these condition 2 TADs are identical to any other TADs in condition 1. Second, the reorganized TAD overlaps with at least two TADs in condition 2, and none of these condition 2 TADs overlap with any other TADs in condition 1. Note that if condition 2 has both TAD(s) identical to the reorganized TAD and TAD(s) overlapping with the reorganized TAD, the classification of the reorganized TAD is based on the exact numbers of identical TADs and overlapping TADs, with the number of identical TADs in condition 2 as the primary criteria and the number of overlapping TADs in condition 2 as the secondary criteria if the primary criteria is inconclusive.
4. *Merge* TADs: *Merge* TADs are the opposite of *split* TADs. Specifically, a reorganized TAD is called a *merge* TAD if it has a many-to-one identical or overlapping relationship with a TAD in condition 2. Specifically, the reorganized TAD satisfies one of the following two criteria. First, the reorganized TAD and at least one of its adjacent/overlapping TADs in condition 1 are identical to a single TAD in condition 2. Second, the reorganized TAD and at least one of its adjacent/overlapping TADs in condition 1 overlap with a single TAD in condition 2.
5. *Zoom* TADs: A reorganized TAD is called a *zoom* TAD if it has a one-to-one overlapping relationship with a TAD in condition 2. Specifically, it satisfies the following three criteria. First, the

reorganized TAD overlaps with exactly one TAD in condition 2. Second, this condition 2 TAD does not overlap with any other TADs in condition 1. Third, the reorganized TAD must not be identical to any TADs in condition 2.

6. *Complex* TADs: All remaining reorganized TADs that do not fit into the previously defined subtypes are classified as *complex* TADs.

Supplementary Method 4 Method comparison

For a fair comparison, we choose alternative methods that calculate P-values: TADCompare (version 1.8.0, installed by Bioconductor) [1], DiffGR (https://github.com/wmalab/DiffGR, commit version 789062e) [2], DiffTAD (https://bitbucket.org/rzaborowski/differential-analysis/src/master/, last updated 2017-02-21) [3], TADsplimer (https://github.com/GuangyWang/TADsplimer, version 1.1) [4], HiCcompare (version 1.20.0, installed by Bioconductor) [5] and HiC-DC+ (version 1.6.0, installed by Bioconductor) [6]. Note that, HiCcompare and HiC-DC+ are designed for detecting differential chromatin interactions. To run both methods, a pseudo chromatin interaction is created for each TAD where the TAD boundaries are the two loci and the sum of Hi-C contact map of the TAD is the Hi-C contact frequency of the pseudo chromatin interaction. We use false positive rate (FPR), true positive rate (TPR) and Jaccard Index (JI) for method evaluation.

FPR (Supplementary Equation 1) is used to quantify performance on controlling false positives (type I errors).

$$\text{FPR} = \frac{\text{FP}}{\text{N}} \tag{1}$$

We assume biological replicates do not have reorganized TADs. FP stands for the number of identified reorganized TADs. N stands for the number of TADs. A lower FPR means a better control of false positives.

TPR (Supplementary Equation 2) is used to quantify performance on identifying truly reorganized TADs (power).

$$\text{TPR} = \frac{\text{TP}}{\text{P}} \tag{2}$$

Here, TP is the number of identified reorganized TADs. P is the number of reorganized TADs in the manually collected gold standard data from a diverse set of publications. Note that a TAD might be counted multiple times if is is reorganized between multiple pairs of conditions. A higher TPR means a higher accuracy in identifying truly reorganized TADs. Combing FPR and TPR, a method with lower FPR and higher FPR is desirable.

JI, $0 \leq \text{JI} \leq 1$, is a metric quantifying the degree of overlap between two sets A and B as defined in Supplementary Equation 3.

$$\text{JI} = \frac{|A \cap B|}{|A \cup B|} \tag{3}$$

JI closer to 1 represents that two sets A and B share more elements. JI is used to compare the sets of identified reorganized TADs by DiffDomain with varied experimental settings. High values of JI indicate that DiffDomain is robust to experimental settings.

Supplementary Method 5 Collection of data used in this study

Bulk Hi-C and scHi-C data

We use the following publicly available data: Hi-C of multiple human cell lines and multiple replicates of GM12878 cell line from Rao et al. [7] (GSE63525 [https://www.ncbi.nlm.nih.gov/geo/query/acc.cgi?acc=GS
down-sampled Hi-C data of GM12878 from Xiong and Ma [8]; Hi-C of patient-derived DIPG, NHA and GBM cell lines and DIPG frozen tissues specimens from Wang et al. [9] (GSE162976 [https://www.ncbi.nlm.nih.gov/

Hi-C of mock-infected and SARS-CoV-2 infected A549-ACE2 cells from Wang et al. [10] (GSE179184 [https://www.ncbi.nlm.nih.gov/geo/query/acc.cgi?acc=GSE179184]). Hi-C of neuronal cell type from Jiang et al. [11] (SRR5617731 [https://www.ncbi.nlm.nih.gov/sra/?term=SRR5617731], SRR5617733 [https://www.ncbi.n and astrocytes from Sofueva et al. [12] (SRR941305, SRR941306 [https://www.ncbi.nlm.nih.gov/sra/?term=SRR941

Processed single-cell Dip-C of multiple cell types in mouse brains are from Tan et al. [13] (GSE162511 [https://www.ncbi.nlm.nih.gov/geo/query/acc.cgi?acc=GSE162511]).

Gold standard reorganized TADs and corresponding Hi-C data

To create gold-standard reorganized TADs, a TAD is called as a reorganized TAD if it meets the following inclusion criteria: (1) The TAD is stated as having structural differences between a pair of conditions in a publication; (2) Chromatin contact map of the TAD is visualized in the main or supplementary figures; (3) Genome coordinate of the reorganized TAD can be explicitly inferred from the publication. In total, the manually collected gold standard data has 65 reorganized TADs that are reported in 15 publications (Supplementary Table 3). For each of such TAD, the Hi-C resolution is one of 5 kb, 10 kb, 25 Kb, 40 kb, 50kb, and 100 kb. Note that we do not choose a universal Hi-C resolution for these gold standard reorganized TADs for two reasons. First, those TADs have highly different lengths (minimum, median, and maximum lengths are 100 kb, 1.3 Mb, and 4.0 Mb, respectively). Second, the Hi-C data have different read depths.

The collected TADs are called as truly reorganized TADs in the main text. These TADs have a varied degree of reorganization and thus a varied difficulty in correctly identifying them. To further testing the performance of DiffDomain and alternative methods, we classify them into different groups. Note that, they are studied in a case-by-case manner and their adjacent TADs are not well described in the original publications. Thus, classifying them through DiffDomain is not suitable. Instead, they are classified according to their original definitions and are broadly categorized into three distinct groups: domain-level change, boundary-level change, and loop-level change. Domain-level change represents widespread changes in interactions within the TAD. Boundary-level change represents TAD reorganization due to changes in TAD boundaries, including boundary strength changes, boundary gain/loos, and boundary shifts. Loop-level change represents that TAD reorganization results from alterations in loops within the TAD, including loop gain/loss or increased/decreased loop contact frequencies. In total, there are 26 pairs of comparisons involving TADs with domain-level changes, 77 pairs involving TADs with boundary-level changes, and 43 pairs involving TADs with loop-level changes.

Epigenomic data for cell lines in Rao et al. [7]

We use the following publicly available data: RNA-seq in K562 from ENCODE[14] (ENCODE: ENCSR000CPZ [https://www.encodeproject.org/experiments/ENCSR000CPZ/]); Super-enhancers of GM12878 and K562 from [15]; DNase peaks and histone modification profiles of multiple cell types from ENCODE[14];

Super-enhancers and oncogenes for NHA and DIPG cell lines

Super-enhancers in NHA, DIPG007, and DIPGXIII are called using ROSE algorithm [16] with input data downloaded from GEO database (GSE162976 [https://www.ncbi.nlm.nih.gov/geo/query/acc.cgi?acc=GSE16297 A super-enhancer is linked to a reorganized TAD if their 1D distance is within 50 kb. Cancer genes are created by intersecting two gene lists: (1) cancer genes downloaded from OncoKB [17]; (2) Genes related to central nervous system that is downloaded from GeneCards with searching keyword [all] (glioma) OR [all] (Pediatric AND Brain AND Tumor) OR [all] (DIPG) OR [all] (central AND nervous AND system). The list of oncogenes is the subset of the above resulted cancer gene list, filtering by the column "Is Oncogene". Oncogenes are then filtered using gene expression TPM > 1, resulting in 61 oncogenes.

Supplementary Method 6 Application to scHi-C data

We briefly describe some methodological details when applying DiffDomain to scHi-C data. Hi-C resolution is chosen at 50 kb for this analysis.

Supplementary Method 6.1 Pseudo-bulk Hi-C data creation and TAD calling

For a given cell type, the pseudo-bulk Hi-C data is created by summing the scHi-C contact matrices from all individual cells with the same cell type. This procedure is used to (1) combine raw scHi-C contact matrices in identifying reorganized TADs between cell types, (2) combine imputed scHi-C contact matrices by scHiCluster in characterizing cell-to-population variability of TADs and cell-to-cell variability of TADs. Similar to TAD calling using bulk Hi-C data in other sections of this paper, once the pseudo-bulk Hi-C data is created, Arrowhead algorithm is applied to call TADs for the cell type. The TAD calling procedure is consistently used in identifying reorganized TADs between cell types, characterizing TADs with differential cell-to-population variability, and characterizing cell-to-cell variability of TADs.

Supplementary Method 6.2 Sampling individual cells to identify reorganized TADs between cell types

Given a cell type with n individual cells, k cells are randomly sampled and used to generate pseudo-bulk Hi-C data (details in Supplementary Method 6.1), $k = 20, 50, \ldots, n$. The sampling procedure is repeated 10 times for each k. The pseudo-bulk Hi-C data with n cells are used in two ways. First, it is used to identify TADs by the Arrowhead algorithm. The resulting TAD list is used consistently for all $k = 20, 50, \ldots, n$. Second, it is used to identify reorganized TADs between a pair of cell types, treated as the gold-standard reorganized TADs for the pair of cell types.

The reproducibility between the set of gold-standard reorganized TADs and the set of reorganized TADs using k sampled cells are quantified using the Jaccard index. A Jaccard index greater than $1/3$ represents that two equal-sized sets share more than half of reorganized TADs.

Supplementary Method 7 Statistical analyses and Hi-C contact map visualization

Statistical analyses were conducted using the R version 3.5.1 and Python version 3.7.3. Hypergeometric test is used to test the enrichment of cancer genes in reorganized TADs. The Wilcoxon test is used to compare difference in means between two distributions such as gene expression, histone modifications and chromatin accessibility. P values smaller than 2.22×10^{-16} are denoted by $P < 2.22 \times 10^{-16}$. Visualization of bulk and single-cell Hi-C contact matrices is done by Nucleome Browser [19] or in-house Python scripts. The APA matrices are based on 25 kb resolution Hi-C data produced by FAN-C using the command 'fanc aggregate -m -p –pixels 90 -r -e –rescale'. The APA plots are generated using python function 'sns.heatmap'.

Supplementary Note 1

Here we show that violation of independence assumption has mild effects on DiffDomain. After computing the normalized difference matrix $\boldsymbol{D}$, DiffDomain reformulates the problem of identifying reorganized TADs into the following hypothesis testing problem:

$$H_0 : \boldsymbol{D}/\sqrt{N} \text{ resembles a generalized Wigner matrix,}$$
$$H_1 : \boldsymbol{D}/\sqrt{N} \text{ does not resemble a generalized Wigner matrix.}$$

A generalized Wigner matrix is a symmetric random matrix with independent mean zero upper diagonal entries. The independence assumption on the upper diagonal entries is violated by $\boldsymbol{D}/\sqrt{N}$ given that Hi-C contact frequencies positively correlate with each other among nearby chromosome bins. Note that DiffDomain does not assume independence among the entries in the individual Hi-C contact matrices.

To investigate the effects of violating the independence assumption on the performance of DiffDomain, we leverage three well-established theoretical results. The first result is the semicircle-law of the empirical spectral distribution of a generalized Wigner matrix $\boldsymbol{A}_{N\times N}$. Specifically, let $\lambda_1 \leq \lambda_2 \leq \cdots \leq \lambda_N$ be the sorted eigenvalues of $\boldsymbol{A}$. The empirical spectral distribution of $\boldsymbol{A}$ is defined as

$$F_N(x) = \frac{1}{N}\sum_{i=1}^{N} \delta_{\lambda_i \leq x}, \tag{4}$$

where δ is an indicator function. As $N \to \infty$, the discrete empirical spectral distribution $F_N(x)$ converges to a continuous distribution function $F(x)$ with the semicircular density function

$$f(x) = \frac{1}{2\pi}\sqrt{(4-x^2)^+}. \tag{5}$$

The second result is the largest eigenvalue λ_N of $\boldsymbol{A}$ converging to 2 in law as $N \to \infty$. The third result is the unadjusted P values following a uniform distribution when H_0 is true and model assumptions are satisfied.

To verify the agreements between empirical properties of $\boldsymbol{D}/\sqrt{N}$ and theoretical properties of generalized Wigner matrices under H_0 and their disagreements under H_1, we choose two sets of real Hi-C data to generate multiple $\boldsymbol{D}/\sqrt{N}$ under H_0 and H_1, respectively. The first set consists of the 8261 GM12878 TADs and two GM12878 Hi-C replicates (*combined* and *primary* in Supplementary Table 1). The 8261 GM12878 TADs should not have much structure reorganization between the GM12878 Hi-C replicates. Thus, the corresponding normalized difference matrices $\boldsymbol{D}_i/\sqrt{N_i}$, $1 \leq i \leq 8261$, are treated as generated under H_0. Second set consists of the manually collected 65 reorganized TADs between pairs of biological conditions from 15 published papers (Supplementary Table 3, Supplementary Method 5). Their normalized difference matrices $\boldsymbol{D}_j/\sqrt{N_j}$, $1 \leq j \leq 146$, are treated as generated under H_1. To verify the distribution of unadjusted P values, we use the P values from investigating TAD reorganization between multiple pairs of the GM12878 Hi-C replicates (Supplementary Table 1) as the P values under H_0 and P values from comparing multiple pairs of human cell lines (Supplementary Table 2) as the P values under H_1.

The analyses find broad agreements between empirical properties of $\boldsymbol{D}/\sqrt{N}$ and theoretical properties of generalized Wigner matrices under H_0 and substantial disagreements under H_1. First, the estimated density functions of empirical spectral distributions of $\boldsymbol{D}_i/\sqrt{N_i}$, $1 \leq i \leq 8261$, generated under H_0 are close to the theoretical semicircular density function (Supplementary Fig. 3a). On the other hand, density functions of empirical spectral distributions of $\boldsymbol{D}_j/\sqrt{N_j}$, $1 \leq j \leq 146$, generated under H_1 have much heavier tails on both sides and thus are different from the theoretical semicircular density function (Supplementary Fig. 3b). Secondly, only a small proportion (12.42%, 1026 out of 8261) of $\boldsymbol{D}_i/\sqrt{N_i}$, $1 \leq i \leq 8261$ generated under H_0 have the largest eigenvalues that are greater than 2 (Supplementary Fig. 3c). In contrast, a large proportion (70.42%, 100 out of 142 possible comparisons) of $\boldsymbol{D}_j/\sqrt{N_j}$, $1 \leq j \leq 146$, generated under H_1 have the largest eigenvalues that are greater than 2 (Supplementary Fig. 3d). Thirdly, the distributions of unadjusted P values under H_0 are approximately flattened over the interval (0, 0.6) (Supplementary Fig. 3e), resembling the expected uniform distribution. The distributions of unadjusted P values under H_1 have peaks around 0 (Supplementary Fig. 3f), indicating the ability of DiffDomain in identifying reorganized TADs. The distributions of P values are much better than those from DiffGR and DiffTAD under H_0 and much better than those HiCcompare and TADCompare under H_1 (Supplementary Fig. 3e,f).

Taken together, these results demonstrate that the normalized difference matrices $\boldsymbol{D}/\sqrt{N}$ from real Hi-C data and generalized Wigner matrices have similar properties under H_0 and different properties

under H_1, highlighting the violation of independence assumption having mild effects on DiffDomain. DiffDomain is appropriate for identifying reorganized TADs using real Hi-C data.

Supplementary Note 2

Supplementary Note 2.1 DiffDomain is robust to sequencing depth

To test the effect of sequencing depth on the performance of DiffDomain, we use down-sampled GM12878 datasets (using 2%, 5%, 10%, and 40% reads). First, we observed that lower the sequencing depth generally resulted in the reduced number of identified reorganized TADs. However, even at 5% reads, DiffDomain still identifies 530 GM12878 TADs that are reorganized in K562. At 40% reads, DiffDomain is able to recover 84.79% of reorganized TADs that are identified using 100% reads. More importantly, we observe that identified reorganized TADs are highly concordant across varied sequencing depths. For example, among the 530 reorganized TADs that are identified using 5% sequencing reads, 524 out of them are also identified as reorganized TADs using 10% sequencing reads (Supplementary Fig. 4a). Similar results are observed when comparing GM12878 data against down-sampled GM12878 data (Supplementary Fig. 4b).

Supplementary Note 2.2 DiffDomain is robust to Hi-C resolution

To test the effect of Hi-C resolution on the performance of DiffDomain, we apply DiffDomain to compare multiple pairs of human cell lines [7]. Hi-C resolution is chosen at 10 kb and 25 kb. We use two metrics to quantify the consistency of DiffDomain at the above two resolutions. The first metric is Jaccard index of two sets. One set is reorganized TADs identified with 10 kb resolution Hi-C data. Another set is reorganized TADs identified with 25 kb resolution Hi-C data. The second metric is the Pearson correlation coefficient. One vector is log-transformed BH-adjusted P values that are computed by DiffDomain with 10 kb resolution Hi-C data. Second vector is log-transformed BH-adjusted P values that are computed by DiffDomain with 25 kb resolution Hi-C data. Overall, Jaccard index and Pearson correlation coefficient are high across multiple pairs of cell types (Supplementary Fig. 4c,d), implying that DiffDomain is robust to a varied Hi-C resolution.

Supplementary Note 2.3 DiffDomain is robust to TAD callers

DiffDomain does not call TADs itself. It leverages multiple TAD callers developed by the community, facilitating commonly performed integrative analysis of Hi-C data and other genomics data. Among the multiple TAD callers, Arrowhead and TopDom are among the recommended TAD callers [18]. Arrowhead identifies hierarchical TADs, i.e., one TAD might contain multiple smaller TADs. In contrast, TopDom identifies non-hierarchical TADs [20]. Thus, Arrowhead and TopDom are chosen to test the robustness of DiffDomain to TAD callers.

Compared with Arrowhead, TopDom identifies a higher number of TADs and identified TopDom TADs cover a higher fraction of the genome across GM12878 replicates and different cell lines (Supplementary Fig. 5a,b). Regardless of the differences in TopDom TADs and Arrowhead TADs, DiffDomain controls the FPRs using both TopDom TADs and Arrowhead TADs (Supplementary Fig. 5c) where FPRs are estimated as the proportions of reorganized TADs between the Hi-C replicates from GM12878 cell line. Note that, when considering Hi-C replicates "GM12878_primary" and "GM12878_replicate" as condition 1 , higher FPRs are observed using Arrowhead TADs than TopDom TADs. This is largely due to higher numbers of Arrowhead TADs than TopDom TADs in both replicates (Supplementary Fig. 5a). Applying to TopDom TADs and Arrowhead TADs in different human cell lines, DiffDomain identifies similar proportions of reorganized TADs between different human cell lines (Supplementary Fig. 5d). These results demonstrate that DiffDomain is robust to TAD callers.

Considering that the hierarchical organization of TADs is a critical feature, we choose the Arrowhead algorithm as the default method to call TADs in this paper.

Supplementary Note 3

We compare DiffDomain with alternative methods in terms of computation time and memory usage. Condition 1 is chosen as GM12878 *combined* Hi-C data which has resolution up to 1 kb. Condition 2 is chosen as GM12878 *primary* Hi-C data, a replicate of GM12878 cell line. GM12878 *combined* Hi-C data has 9275 TADs that are identified by Arrowhead. For a fair comparison, computation time is estimated as CPU time (*user*) and memory usage is estimated as *memory resident set size*. Both statistics are estimated by Linux kernel function */usr/bin/time -f "%M %e"*. The experiment is repeated 10 times and the average is reported. We find that DiffDomain consistently uses the least memory across the varied Hi-C resolution compared with other methods (Supplementary Fig. 12b), demonstrating that DiffDomain is memory efficient. The reason is that DiffDomain iteratively compares Hi-C contact matrices of TADs, only loading Hi-C contact matrices for a given TAD into RAM. In contrast, other methods require loading Hi-C contact matrices of a whole chromosome into RAM. Memory efficiency does not make DiffDomain the slowest in computation time. DiffDomain completes the comparison of 9275 TADs within 1275 seconds (21.25 minutes) under commonly used Hi-C resolutions (10 kb, 25 kb, and 50 kb), which is much faster than DiffGR (Supplementary Fig. 12). Importantly, unlike other methods, DiffDomain has stable memory usage and computation time (Supplementary Fig. 12). Together, these results demonstrate that DiffDomain is memory efficient and reasonably fast.

Supplementary Note 4

Here we show that reorganized TADs are associated with changes in numbers of CTCF peaks. Earlier studies show that TAD boundaries are enriched with CTCF peaks [21]. To further demonstrate the biological relevance of reorganized TADs identified by DiffDomain, we compare the number of CTCF peaks in the boundaries of reorganized TADs and the other TADs. Regarding the reorganized IMR90 TADs in K562, reorganization of IMR90 TADs in K562 results in three types of TAD boundaries: lost, new, and stable. For simplicity, the K562 TAD boundaries that do not coincide with these TAD boundaries are called as the other TAD boundaries in K562 (Supplementary Fig. 14a). First, lost IMR90 TAD boundaries in K526 are due to *loss*, *zoom*, and *merge* subtypes of reorganized TADs. These TAD boundaries have significantly ($P \leq 2.22 \times 10^{-16}$) fewer CTCF peaks than the other TAD boundaries in K562. Second, new K562 TAD boundaries are created in K562 due to *split* and *zoom* subtypes of reorganized TADs. These TAD boundaries have no significant difference in the number of CTCF peaks compared with the other TAD boundaries in K562. Thirdly, stable TAD boundaries are TAD boundaries of *strength-change* subtype of reorganized TADs. The subset of these TAD boundaries that are from *strength-change* subtype of reorganized TADs with increased contact frequencies (*strength-change up TADs*) have significantly ($P \leq 2.22 \times 10^{-16}$) higher number of CTCF peaks than the other K562 TADs. In contrast, the subset of these TAD boundaries that are from *strength-change* subtype of reorganized TADs with decreased contact frequencies has no significant difference in the number of CTCF peaks compared with the other K562 TADs. Importantly, *complex* subtype of reorganized TADs have both lost and new TAD boundaries in K562. These TAD boundaries have no significant difference in the number of CTCF peaks compared with the other K562 TAD boundaries. Note that due to complexity and a small proportion of *complex* reorganized TADs, we do not stratify the comparison by lost and new types of TAD boundaries (Supplementary Fig. 14a). Repeating the analysis to other pairs of cell types find similar patterns (Supplementary Fig. 14b). These observations are consistent with the enrichment of CTCF

peaks at TAD boundaries [21]. Additionally, a *merge* TAD represents two or more TADs of condition 1 are merged into one TAD in condition 2. In a *merge* TAD, the boundaries that are lost in condition 2 have fewer CTCF peaks in condition 2. The observation is consistent with the observation by Wang et al. [4], in which TAD splits and TAD merges between a pair of conditions are investigated. These results demonstrate that the reorganized TADs identified by DiffDomain are reasonable and biologically relevant.

Supplementary Note 5

Here we show that proportion of reorganized TADs is consistent with cell type identities. Armed with superior performance in terms of both FPR and TPR, we next apply DiffDomain to real data sets for biological relevance analyses. First, we investigate the TAD structural changes between different biological conditions by applying DiffDomain to 7 different human cell types (Supplementary Table 2). The proportions of reorganized TADs between cell types are reported in Supplementary Fig. 7. Overall, TADs are stable across cell types. Among the 42 pairwise combinations between the 7 cell types, the median proportion of significantly reorganized TADs is 13.76%. The observation is consistent with the previous study [21]. On the other hand, we do find that the proportion of reorganized TADs has a high variety and is cell type dependent. For example, the K562 TADs have the smallest proportion (11.41%) of reorganized TADs in KBM7 (both K562 and KBM7 are chronic myeloid leukemia). The highest proportion (41.24%) of reorganized K562 TADs is found in IMR90, a normal cell type. The ratio of the highest proportion to the smallest proportion is 3.61. Repeating the analysis to the other cell types, we find that the ratios are no less than 2.74. Similar results are observed at 25 kb resolution (Supplementary Fig. 7). Hierarchical clustering reveals that cell types with similar developmental relationships have smaller proportions of reorganized TADs (Supplementary Fig. 15), further demonstrating the utilization of DiffDomain.

Supplementary Note 6

Here we show that reorganized TADs are enriched in cancer genes and associated with epigenomic changes.

First, we compare human hematopoietic cells: K562 (chronic myeloid leukemia cell lines) and GM12878 (normal lymphoblastoid cell line) cell lines. We downloaded 128 cancer genes that are related to chronic myelogenous leukemia from GeneCards [22]. GM12878 TADs that are reorganized in K562 are enriched (hypergeometric test, P=0.01, 66 out of the 128 cancer genes) with the cancer genes, indicating disease-trait relevance of reorganized TADs.

Second, we investigate the association between reorganized TADs and chromatin accessibility. Among the reorganized GM12878 TADs in K562, 16.25% of them have at least a 2-fold increase in DNase peak coverage in K562 cell type. Generally, the reorganized GM12878 TADs have significantly ($P < 2.22 \times 10^{-16}$) higher fold-change in DNase peak coverage in K562 than the other GM12878 TADs. The pattern for GM12878 TADs is consistently observed in the other cell types. Similar patterns are also observed for HUVEC and K562 TADs (Supplementary Fig. 16). These results suggest that the reorganized TADs tend to gain chromatin accessibility. Noticeably, we found reverse patterns for HMEC TADs. Compared to the other HMEC TADs, the reorganized HMEC TADs have significantly higher loss in DNase peaks in GM12878, HUVEC, and K562 cell types. Taken together, our results suggest that structural changes in TADs are associated with gain/loss of chromatin accessibility in the TAD regions across cell types.

We further ask the connection between TAD structural rewiring and histone modification dynam-

ics in the TAD. It is known that histone modifications of H3K27ac and H3K4me1 are markers of active/poised enhancers and super-enhancers. H3K4me3 and H3K36me3 mark active/poised promoters and actively transcribed regions, respectively [23–25]. We find that reorganized TADs have significantly higher fold-changes in H3K27ac and H3K4me1/2 signals comparing with the other TADs. The significance patterns are much more profound than the patterns for H3K4me3 and H3K36me3 signals (Supplementary Fig. 17). In the subsets of TADs with gain (fold-change > 1) in chromatin accessibility or histone modifications signals, TAD reorganization subtypes *strength-change up*, *zoom*, *split*, and *complex* have significantly higher proportion of TADs with at least 2-fold gain in DNase peak coverage, H3K27ac, H3K4me1, and H3K4me3 signals (Supplementary Fig. 18a). In contrast, within the subsets of TADs with decrease (fold-change < 1) in chromatin accessibility or histone modifications signals, TAD reorganization subtypes *loss*, *strength-change down* and *merge* have significantly higher proportions of TADs with at least 2-fold loss in H3K27ac, H3K4me1, and H3K4me3 signals (Supplementary Fig. 18b). These results underscore distinct associations between TAD reorganization subtypes and chromatin accessibility as well as histone modifications. Specifically, TAD reorganization subtypes *strength-change up*, *zoom*, *split*, and *complex* are associated with increased chromatin accessibility and histone modifications signals marking active transcription activities. Conversely, TAD reorganization subtypes *loss*, *strength-change down* and *merge* are associated with decreased histone modifications signals marking active transcription activities, emphasizing the importance of TAD reorganization subtypes in investigating genome activity and functionality. The findings are consistent with previous study [25]. The authors discovered that histone modifications on promoters are stable across cell types. While the histone modifications on enhancers are cell type-specific and strongly associated with cell type-specific gene expressions. The findings are also consistent with the findings that *split* and *merge* subtypes of reorganized TADs are associated with changes in chromatin epigenetic state [4].

To summarize, comparative epigenomic analyses reveal that structurally reorganized TADs are enriched in cancer genes, and have associated changes in epigenomic profiles, suggesting the biological relevance of reorganized TADs.

Supplementary Note 7

Here we show that reorganized TADs are biologically relevant to diffuse intrinsic pontine glioma (DIPG).

DIPG is a pediatric high-grade glioma that is the leading cause of cancer death in children. A recent study generates the first high-resolution Hi-C data of DIPG cell lines and reveals that reorganized TADs are associated with alternations of transcriptional regulation [9]. However, the study uses a boundary-based method to identify reorganized TADs, missing reorganized TADs without boundary changes (*strength-change* TADs) and lacking statistical significance as mentioned in the Introduction section. To demonstrate the utilization of DiffDomain, we reanalyze the data.

We first focus on the NHA TAD that harbors the oncogenes MYCN. The TAD is identified as a *split* TAD in DIPG cell line DIPG007 and as a *zoom* TAD in DIPG cell line DIPGXIII (Supplementary Table 4), consistent with the previous observations that chromatin interactions in the TAD are strengthened in DIPG007 and DIPGXIII (Fig. 3a in [9]). Genome-wide, DiffDomain identifies that 14.89% (352) and 15.78% (373) of NHA TADs are reorganized in DIPG007 and DIPGXIII, respectively. The two sets of reorganized TADs share more than 73.46% of reorganized TADs (Supplementary Fig. 19a), consistent with the fact that both DIPG007 and DIPGXIII are patient-derived cell lines. Among the reorganized TADs, 26.99% and 26.27% are *strength-change* TADs (Supplementary Fig. 20a,c), refining the TAD reorganization analysis as reported in Wang et al. [9]. Although these reorganized TADs are not significantly enriched in oncogenes in DIPG007 and DIPGXIII, multiple subtypes of reorganized

TADs including *strength-change* TADs, *split* TADs, and *merge* TADs, harbor at least one oncogenes (Supplementary Table 4). Four examples are visualized in Supplementary Fig. 21. Among the subsets of oncogenes (Supplementary Table 4), *MYCN*, *CCND1* genes are well-known functional genes in DIPG; *SOX2*, *VEGFA* and other oncogenes are under-explored in DIPG, which may have therapeutic implications for DIPG. Stratifying the results by super-enhancers, two reorganized TADs harbor both oncogenes and DIPG007 super-enhancers (Supplementary Method 5), two reorganized TADs harbor both oncogenes and DIPGXIII super-enhancers (see Supplementary Fig. 21c,d for two examples), expanding the analysis from enhancers [9] to super-enhancers. Additionally, a higher number of DIPG007-DMSO TADs are reorganized after the BRD degrader treatment (dBET6) compared with BET BRD inhibitor treatment (BRD4i) (41 in dBET6 vs. 9 in BRD4i, 5 are shared, 10 kb Hi-C resolution, Supplementary Fig. 19d). The preliminary results suggest stronger effects of dBET6 on TADs, consistent with dBET6 having stronger effects on chromatin loops and A/B compartments [9]. Hi-C resolution at 10 kb and 25 kb provide similar results (Supplementary Fig. 19b,c, 20b,d). These results further demonstrate the utilization of DiffDomain and the functional relevance of identified reorganized TADs.

Supplementary Note 8

Here we show that reorganized TADs are associated with epigenome reprograme after SARS-CoV-2 infection. Regarding epigenome reprogramme, the reorganized TADs have significantly ($P < 2.22 \times 10^{-16}$) higher numbers of enhanced and weakened peaks of H3K27ac (Fig. 5c, Supplementary Fig. 26c), a marker for active enhancers. The reorganized TADs have significantly ($P < 2.22 \times 10^{-16}$) higher numbers of enhanced and weakened peaks of SMC3 (Fig. 5d, Supplementary Fig. 26d), a cohesin subunit that regulates 3D genome organization. Similar patterns are observed for another key cohesin subunit RAD21 (Fig. 5e, Supplementary Fig. 26d), but to a lesser degree than SMC3 because a much smaller number of differential peaks are called by MAnorm2. Stratifying the above comparative analysis by the subtypes of reorganized TADs, similar patterns are observed (Fig. 5c-e, Supplementary Fig. 26c-e), demonstrating that enrichments of differential peaks of H3K27ac, SMC3, and RAD21 have no strong preference over the subtypes of reorganized TADs.

Supplementary Note 9

Here we show that distinct associations of differentially expressed genes with differential chromatin interactions in reorganized TADs after SARS-CoV-2 infection. To explore the connection between differentially expressed genes and their potential differential Hi-C interactions, we use HiC-DC+ to identify differential chromatin interactions. HiC-DC+ is run using Hi-C resolution of 10 kb, ssiz of 0.1, and other parameters with default values. Given the relatively low resolution of Hi-C data, only a limited number of significantly differential chromatin interactions are identified with an adjusted P value threshold of 0.05, Thus, the original P value with threshold of 0.05 is used to identify significantly differential chromatin interactions. First, we study down-regulated genes. A down-regulated gene is called as having a differential chromatin interaction if its transcription start site is located within 50 kb of one of the chromosome bins involved in the differential chromatin interaction. We find that, among the down-regulated genes located in reorganized TADs, 36.9% (62 out of 168) have at least one differential chromatin interactions (Supplementary Fig. 27a). The proportion is 1.74 times higher than that of the down-regulated genes located in the other TADs (21.2%, 92 out of 433, Supplementary Fig. 27a). Due to low number of differential chromatin interactions in down-regulated genes (Supplementary Fig. 27b,c), down-regulate genes are classified into four groups depending on involved differential chromatin interactions: with only enhanced chromatin interaction, with only weakened chromatin interaction, with both enhanced

and weakened chromatin interaction, and without differential chromatin interaction. Stratified by the group of down-regulated genes and subtype of reorganized TADs, comparative analysis reveals that down-regulated genes located in *strength-change* TADs have a 3 times higher proportion (9.73%) of down-regulated genes with both enhanced and weakened chromatin interactions compared with down-regulated genes located in the other TADs (Supplementary Fig. 27f). Down-regulated genes in the other subtypes of reorganized TADs also have distinct patterns involving differential chromatin interactions compared to down-regulated genes in the other TADs (Supplementary Fig. 27f). Similar observations are observed when analyzing up-regulated genes (Supplementary Fig. 27a,d,e,g). These observations underscore the role of TAD reorganization in gene expression regulation.

Supplementary Tables

Supplementary Table 1: Summary of the multiple Hi-C replicates from GM12878 cell line [7]. The replicates are produced with different settings and have different total Hi-C contacts. The highest total Hi-C contacts (combined) is 18.9 fold higher than the lowest total Hi-C contacts (noXlink). The data are used to quantify the false positive rates of DiffDomain and alternative methods.

Abbreviation	Restriction enzyme	Crosslinking	# contacts
Primary	MboI	Yes	3,587,190,419
Replicate	MboI	Yes	2,937,330,058
Combined	MboI	Yes	6,524,520,477
DpnII	DpnII	Yes	567,550,645
noXlink	MboI	No	345,058,337

Supplementary Table 2: Brief description of the seven cell types [7] used in the study. Two of them are cancer cell lines.

Cell line	Disease	Cell type	# contacts
GM12878	Normal	B-lymphoblastoids	6,524,520,477
HMEC	Normal	Mammary Epithelial	538,392,678
HUVEC	Normal	Umbilical Vein Endothelial	732,108,988
IMR90	Normal	Lung Fibroblasts	1,535,222,082
NHEK	Normal	Epidermal Keratinocytes	1,073,207,572
K562	Cancer	Erythroleukemia	1,366,228,845
KBM7	Cancer	Near Haploid Human Myelogenous Leukemia	1,247,936,408

Supplementary Table 3: Gold-standard reorganized TADs that are manually collected from multiple studies.

N	Reference	TAD region	GEO	Species
1	Yang et al. [26]	Chr11:33750000:35600000	GSE134055	Zebrafish
2		Chr13:10000000:11800000		
3		Chr4:94880000:96040000		
4	Goodman et al. [27]	Chr10:117390000:117760000	GSE138822	Mouse
5		Chr13:28260000:29080000		

6		Chr2:93300000:93455000		
7	Huang et al. [28]	Chr3:34601078:34811078	GSE153403	Mouse
8		Chr18:54000000:56000000		
9		Chr1:136500000:138000000		
10		Chr1:68000000:70000000		
11	Szabo et al. [29]	Chr2:138000000:139500000	GSE96107	Mouse
12		Chr2:86500000:88500000		
13		Chr8:41500000:44000000		
14		Chr2:55000000:57000000		
15	Lee and Roy [30]	ChrX:162000000:164250000	GSE96107	Mouse
16		Chr12:26000000:29000000		
17		Chr13:27500000:30500000		
18		Chr2:49900000:52400000		
19		Chr3:100800000:102400000		
20		Chr3:33000000:35700000		
21	Bonev et al. [31]	Chr4:22300000:24300000	GSE96107	Mouse
22		Chr6:51100000:52400000		
23		Chr8:42100000:44500000		
24		Chr8:53800000:56600000		
25		Chr9:101500000:102800000		
26		Chr5:15900000:16000000		
27	Winick-Ng et al. [32]	chr18:34650000:35850000	GSE148792	Mouse
28		chr18:36000000:39000000		
29	Xiao et al. [33]	Chr11:2200000:2500000	GSE143937	Human
30		Chr11:1500000:2200000		
31		Chr1:159730485:160395186		
32		Chr2:212000000:213481530		
33		Chr5:172979739:173460858		
34		Chr7:20572855:22172668		
35	Zhang et al. [34]	Chr14:35850000:36478806	GSE137376	Human
36		Chr2:13142351:14400000		
37		Chr6:101546941:103132463		
38		Chr6:74460000:76260000		
39		Chr10:59720000:61320000		
40	Bertero et al. [35]	Chr13:75000000:76400000	GSE106690	Human
41		Chr21:32900000:33150000		
42		Chr8:118070000:118430000		
43		Chr18:59350000:59950000		
44	Wang et al. [9]	Chr2:15600000:16800000	GSE162976	Human
45		Chr12:8430000:9210000		
46		Chr11:15570000:16650000		
47		Chr21:32990000:33135000		

48	Xing et al. [36]	Chr2:40000000:41000000	GSE63525	Human
49		Chr1:54280000:55280000		
50		Chr1:55280000:57040000		
51		Chr1:58880000:60120000		
52	Dixon et al. [37]	Chr1:63040000:63800000	GSE52457	Human
53		Chr1:63800000:64480000		
54		Chr1:60120000:61080000		
55		Chr1:62400000:62680000		
56		Chr10:53400000:54500000		
57		Chr5:11400000:13500000		
58		Chr5:24600000:26400000		
59		Chr13:54500000:58500000		
60	Zhang et al. [38]	Chr14:37700000:39700000	GSE116862	Human
61		Chr4:91000000:95000000		
62		Chr4:91912127:94698321		
63		Chr13:55801210:58182661		
64		Chr20:17500000:18100000		
65	Rowley et al. [39]	Chr1:163500000:165000000	GSE63525	Human

Supplementary Table 4: Oncogenes in the reorganized TADs when comparing NHA with DIPG cell lines (DIPG007 and DIPGXIII). Abbreviation: NHA, normal human astrocytes; DIPG, pediatric high-grade glioma.

Comparison	Reorganized TAD	Subtype of reorganization	Oncogene
	chr2:15640000-16580000	*split*	*MYCN*
	chr3:18170000-182690000	*split*	*SOX2*
	chr3:189250000-189950000	*strength-change*	*TP63*
NHA vs. DIPG007	chr6:43760000-44080000	*loss*	*VEGFA*
	chr8:127710000-129770000	*zoom*	*MYC*
	chr11:69100000-69660000	*loss*	*CCND1*
	chr2:15640000-16580000	*zoom*	*MYCN*
	chr3:181700000-182690000	*zoom*	*SOX2*
	chr3:189250000-189950000	*strength-change*	*TP63*
	chr6:43630000-44080000	*loss*	*VEGFA*
NHA vs. DIPGXIII	chr7:54760000-55250000	*strength-change*	*EGFR*
	chr8:127710000-129770000	*split*	*MYC*
	chr10:120940000-121720000	*strength-change*	*FGFR2*
	chr11:69100000-69660000	*merge*	*CCND1*
	chr12:102100000-102770000	*strength-change*	*IGF1*

Supplementary Table 5: Summary of SVs and Hi-C data used for analyzing the associations between SVs and reorganized TADs identified by DiffDomain. SVs detected by the deep learning method EagleC using bulk and scHi-C data are downloaded from Wang et al. [40]. Condition 1 contains GM12878 and NHA cell lines, representing health conditions. Condition 2 contains K562, DIPG007, and DIPGXIII cell lines, representing disease conditions.

N	Condition 1	Condition 2	# SVs associated with condition 1 TADs	Hi-C data (Ref.)
1	GM12878	K562	39	[7]
2	NHA	DIPG007	36	[9]
3	NHA	DIPGVIII	23	[9]

Supplementary Figures

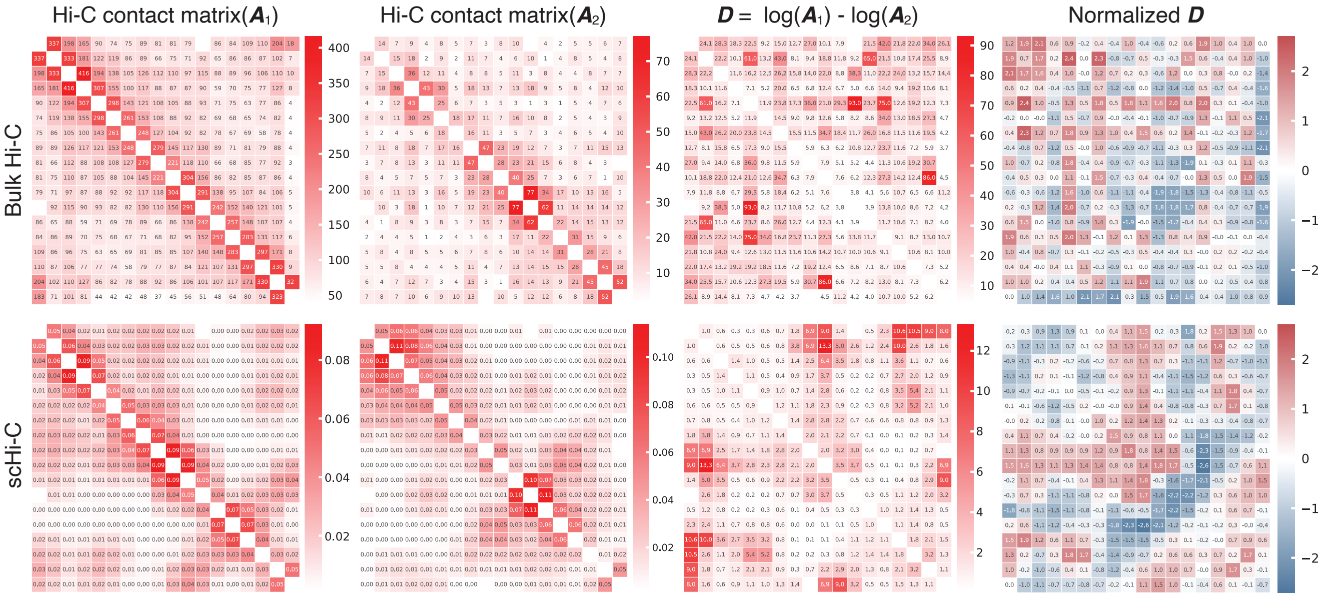

Supplementary Figure 1: Visualization of the procedures in DiffDomain for both bulk and scHi-C data as the input for the same TAD. *Top;* bulk Hi-C contact matrices as the input ($\boldsymbol{A}_1$ and $\boldsymbol{A}_2$); *Bottom* scHi-C contact matrices imputed by scHiCluster as the input ($\boldsymbol{A}_1$ and $\boldsymbol{A}_2$). Cortical L2-5 pyramidal cells are condition 1. Adult astrocytes are condition 2. The TAD region is Chr3:128,350,000:129,200,000. *First* column is the input contact matrix from condition 1. *Second* column is the input contact matrix from condition 2. *Third* column is log-transformed difference matrix $\boldsymbol{D}$. *Forth* column is normalized $\boldsymbol{D}$ by iteratively standardizing its k-off diagonal elements, $-(N-1) \leq k \leq N-1$. Although the same TAD is used, the normalized $\boldsymbol{D}$ in the two rows have some differences largely due to high randomness of TAD organization in individual cells. In both bulk Hi-C and scHi-C scenarios, the TAD is identified as a reorganized TADs. This visualization is an addition to the visualization in Fig. 1a-c, highlighting that DiffDomain can work on both bulk and single-cell Hi-C data.

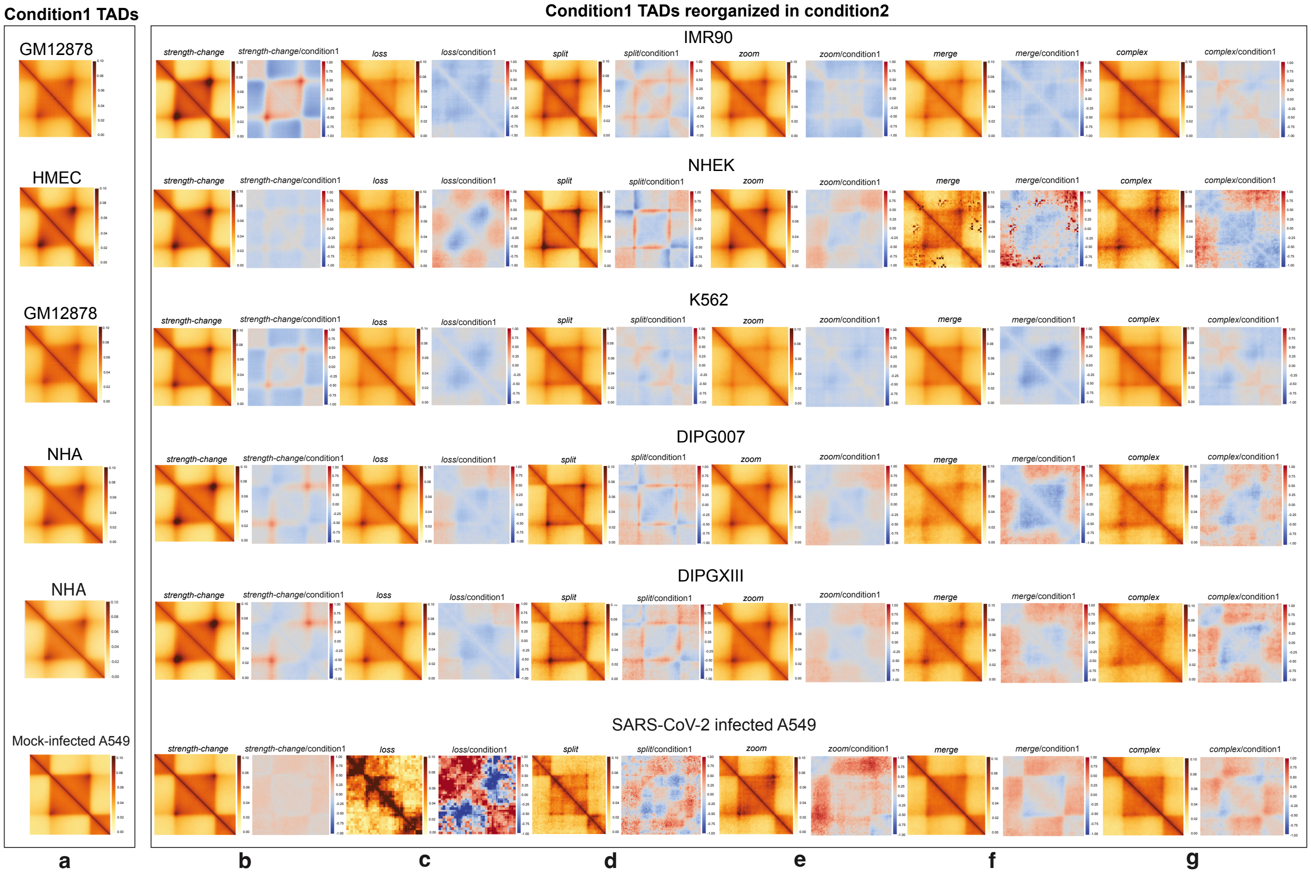

Supplementary Figure 2: APA plot summarizing subtypes of reorganized TADs. **a** APA plot summarizing all TADs in condition 1 using Hi-C data from the same condition. The APA plot is consistently used as a control in the subsequent APA plots to highlight aggregated changes in the subtypes of reorganized TADs. It is referred to as condition 1 in the subsequent APA plots. **b** APA plot summarizing aggregated changes in *strength-change* TADs. *Left* APA plot for *strength-change* TADs using Hi-C data from condition 2; *Right* APA plot showcasing log2-transformed fold-change of APA matrices, where the numerator is from the *Left* and the denominator is from **a**. Decreased/increased interactions within the aggregated TAD region alongside stable interactions around its boundaries support *strength-change* TAD definition. **c** APA plot summarizing aggregated changes in *loss* TADs. Decreased interactions within the aggregated TAD region and around its boundaries align with *loss* TAD characterization. **d** APA plot summarizing aggregated changes in *split* TADs. Decreased interactions within the aggregated TAD region, particularly around its center, is consistent with the definition of *split* TADs. **e** APA plot summarizing aggregated changes in *zoom* TADs. Increased/decreased interactions within the aggregated TAD region and between it and adjacent upstream/downstream regions are typical of *zoom* TADs. **f** APA plot summarizing aggregated changes in *merge* TADs. Increased interactions between the aggregated TAD region and its adjacent regions align with *merge* TAD definition. **g** APA plot summarizing aggregated changes in *complex* TADs. Across the six pairs of conditions (*Rows*), distinct changes within the aggregated TAD region and between it and its adjacent regions match *complex* TAD description. These pairs of conditions include two pairs of normal cell lines (GM12878 vs. IMR90, HMEC vs. NHEK) and four pairs of normal cell lines and cell lines with disease (GM12878 vs. K562, NHA vs. DIPG007, NHA vs. DIPGXIII, mock-infected vs. SARS-CoV-2 infected A549-ACE2 cells). The APA matrices are based on 25 kb resolution Hi-C data produced by FAN-C using the command ‘fanc aggregate -m -p –pixels 90 -r -e –rescale’. The APA plots are generated using python function ‘sns.heatmap’.

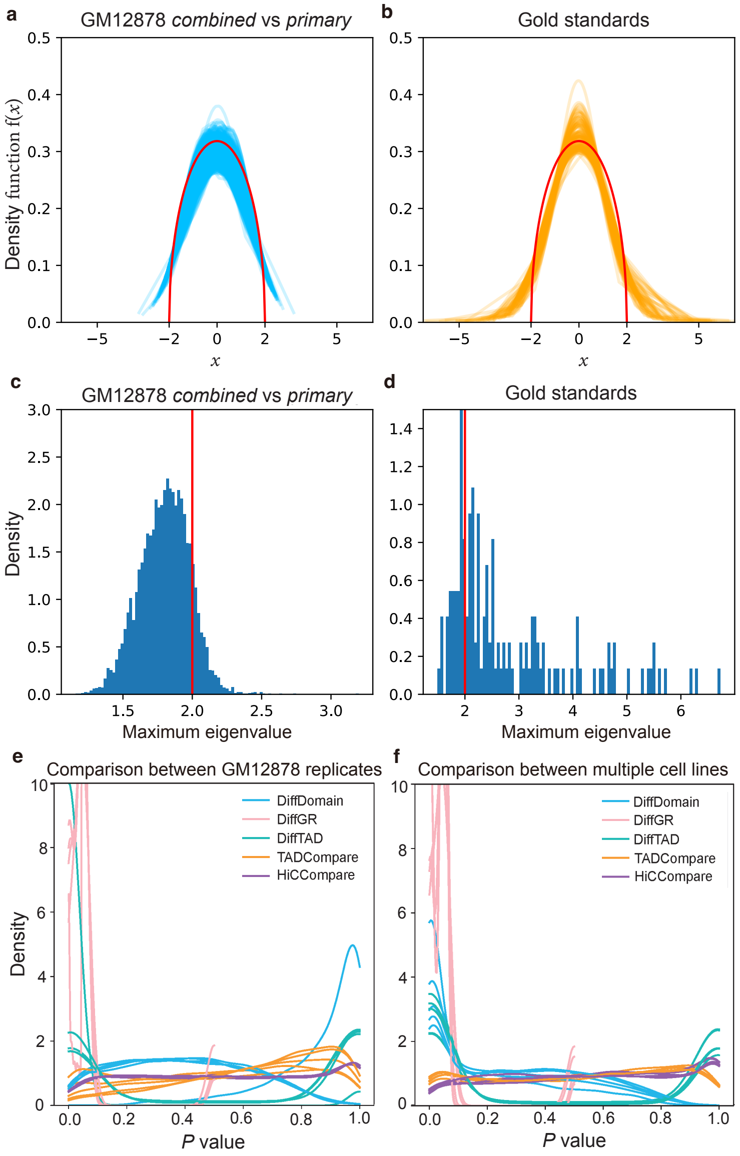

Supplementary Figure 3: Violation of the independence assumption by $\boldsymbol{D}/\sqrt{N}$ of DiffDomain has modest effects. **a** Estimated density functions $f_i(x)$ of empirical spectral distributions of $\boldsymbol{D}_i/\sqrt{N_i}$, $1 \leq i \leq 8261$, that are generated using GM12878 TADs and two GM12878 Hi-C replicates. Those $\boldsymbol{D}_i/\sqrt{N_i}$, $1 \leq i \leq 8261$, are treated as generated under H_0. Red curve is the theoretical semicircular density function of generalized Wigner matrices under H_0. **b** Estimated density functions $f_j(x)$ of empirical spectral distributions of $\boldsymbol{D}_j/\sqrt{N_j}$, $1 \leq j \leq 146$, that are generated using manually collected 65 reorganized TADs. Those $\boldsymbol{D}_j/\sqrt{N_j}$, $1 \leq j \leq 146$, are treated as generated under H_1. **c** Histogram shows the largest eigenvalues of $\boldsymbol{D}_i/\sqrt{N_i}$, $1 \leq i \leq 8261$, same as in **a**. The vertical red line is the theoretical limit of the largest eigenvalue of a generalized Wigner matrix (H_0). **d** Histogram shows the largest eigenvalues of $\boldsymbol{D}_j/\sqrt{N_j}$, $1 \leq j \leq 146$, same as in **b**. **e** Estimated density curves of unadjusted P values that are computed in investigating TAD reorganization between multiple pairs of the GM12878 Hi-C replicates (Supplementary Table 1). Each curve corresponds to the comparison between a pair of GM12878 Hi-C replicates. The unadjusted P values are treated as the P values under H_0. P values follow a uniform distribution when H_0 is true and model assumptions are satisfied. **f** Estimated density curves of unadjusted P values that are computed in comparing multiple pairs of human cell lines (Supplementary Table 2). The unadjusted P values are mixtures of P values under H_1 and P values under H_0, thus they are not uniformly distributed, as expected. DiffDomain assumes that $\boldsymbol{D}/\sqrt{N}$ is a generalized Wigner matrix, a symmetric random matrix with independent upper diagonal entries. The independence assumption on the upper diagonal entries is violated by $\boldsymbol{D}/\sqrt{N}$ given that Hi-C contact frequencies positively correlate with each other among nearby chromosome bins. The analyses find broad agreements between empirical properties of $\boldsymbol{D}/\sqrt{N}$ and theoretical properties of generalized Wigner matrices under H_0 and substantial disagreements under H_1, showing violation of independence assumption of DiffDomain has modest effects.

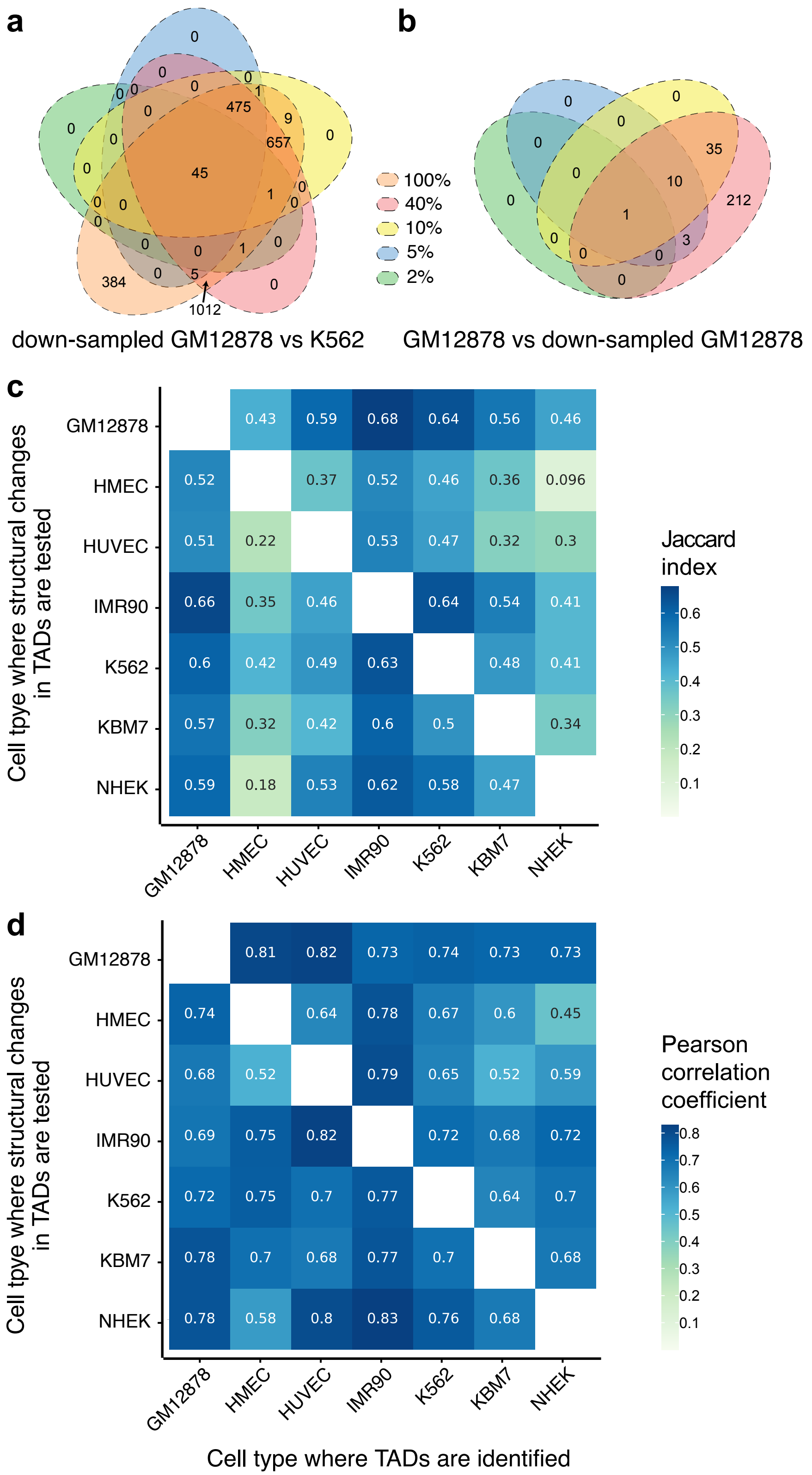

Supplementary Figure 4: DiffDomain is robust to sequencing depths and Hi-C resolution. **a** Venn diagram showing reproducibility of reorganized TADs that are identified by DiffDomain using a varied sequencing depth. **b** Venn diagram showing reproducibility between original and down-sampled Hi-C data from GM12878. Sequences were down-sampled at $2, 5, 10, 40\%$ sequencing reads. **c** Heatmap showing Jaccard index between reorganized TADs that are identified by DiffDomain using 10 kb and 25 kb resolution Hi-C data. **d** Heatmap showing Pearson correlation coefficient between log-transformed BH-adjusted P values. One list of P values is computed by DiffDomain using 10 kb resolution Hi-C data. Another list of P value is computed using 25 kb resolution Hi-C data.

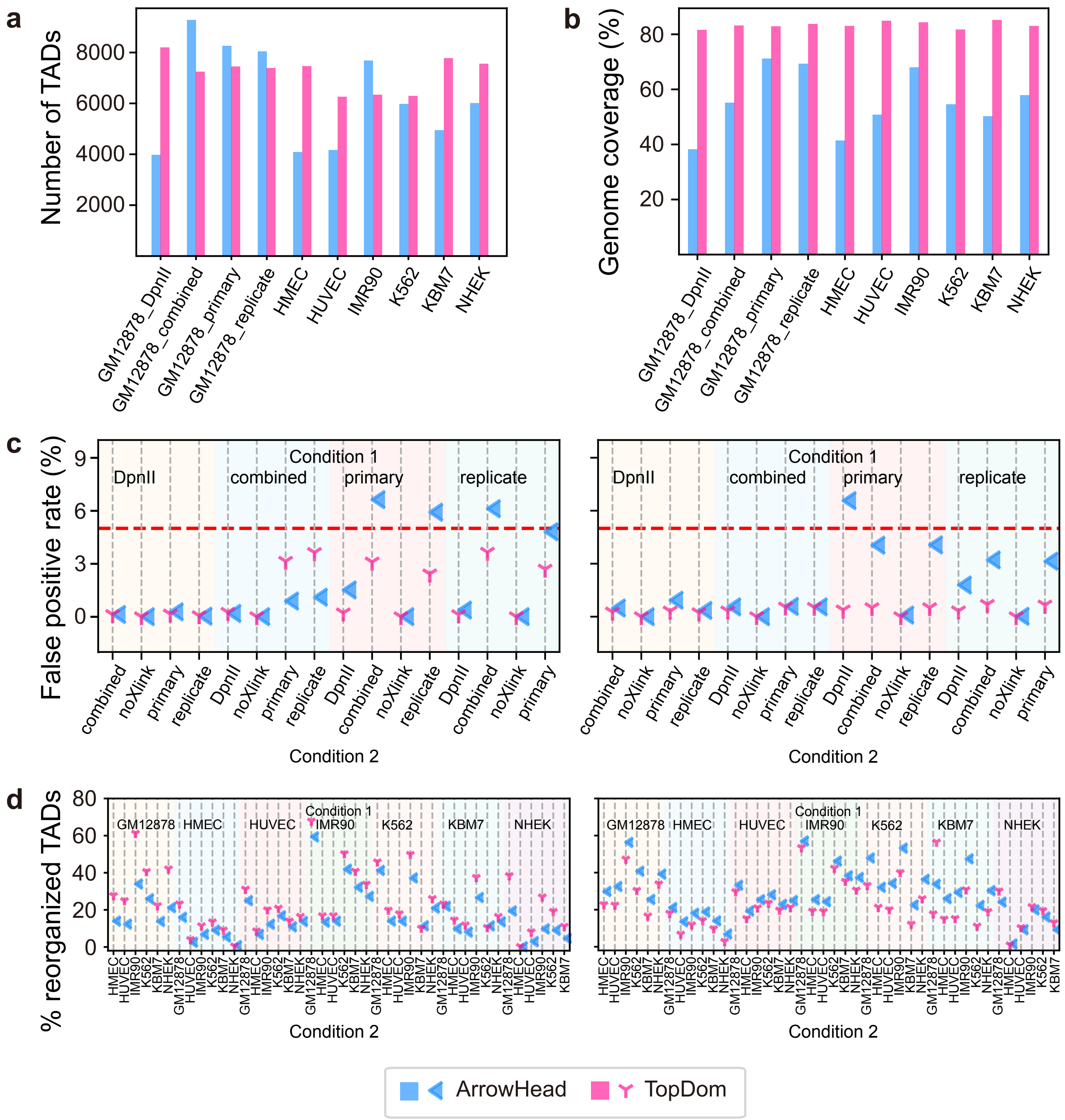

Supplementary Figure 5: DiffDomain is robust to TAD callers. **a** Bar graph showing the number of TADs called by Arrowhead and TopDom across GM12878 replicates and different cell lines. **b** Bar graph showing the genome coverage of TADs. **c** Dot plot showing the false positive rates of DiffDomain. False positive rate is estimated as the proportion of reorganized TADs between GM12878 replicates. Hi-C resolution is 10 kb and 25 kb, respectively. **d** Dot plot showing the proportion of reorganized TADs between different human cell lines. Hi-C resolution is 10 kb and 25 kb, respectively. Overall, although the TADs called by Arrowhead and TopDom have clear differences in the number of TADs and genome coverage, DiffDomain has comparable FPRs and proportions of reorganized TADs between cell lines, suggesting that DiffDomain is robust to TAD callers.

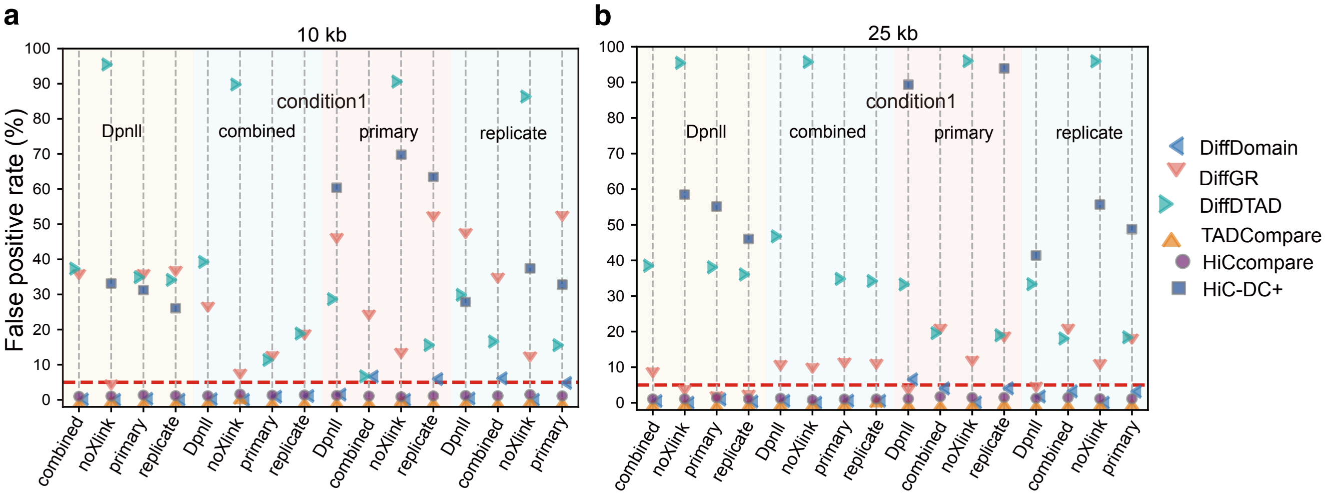

Supplementary Figure 6: Method comparison using FPR. The resolution of Hi-C data are 10 kb **a** and 25 kb **b**. Given a pair of GM12878 Hi-C replicates, FPR is estimated as the percentage of identified reorganized GM12878 TADs. The GM12878 Hi-C replicate "noXlink" as condition 1 is missing because it does not have enough read coverage to call TADs by Arrowhead algorithm at 10 kb resolution. The horizontal dashed red lines represent significant level $\alpha = 0.05$, which is also the expected FPR under H_0. Note that HiC-DC+ requires at least two Hi-C replicates in each condition. The Hi-C replicate *combined* is created by merging the replicates *primary* and *replicate*. Thus, HiC-DC+ is not applied to compare replicate *combined* with other replicates. These results show that DiffDomain, TADCompare, and HiCcompare have better control of FPR than the other three methods.

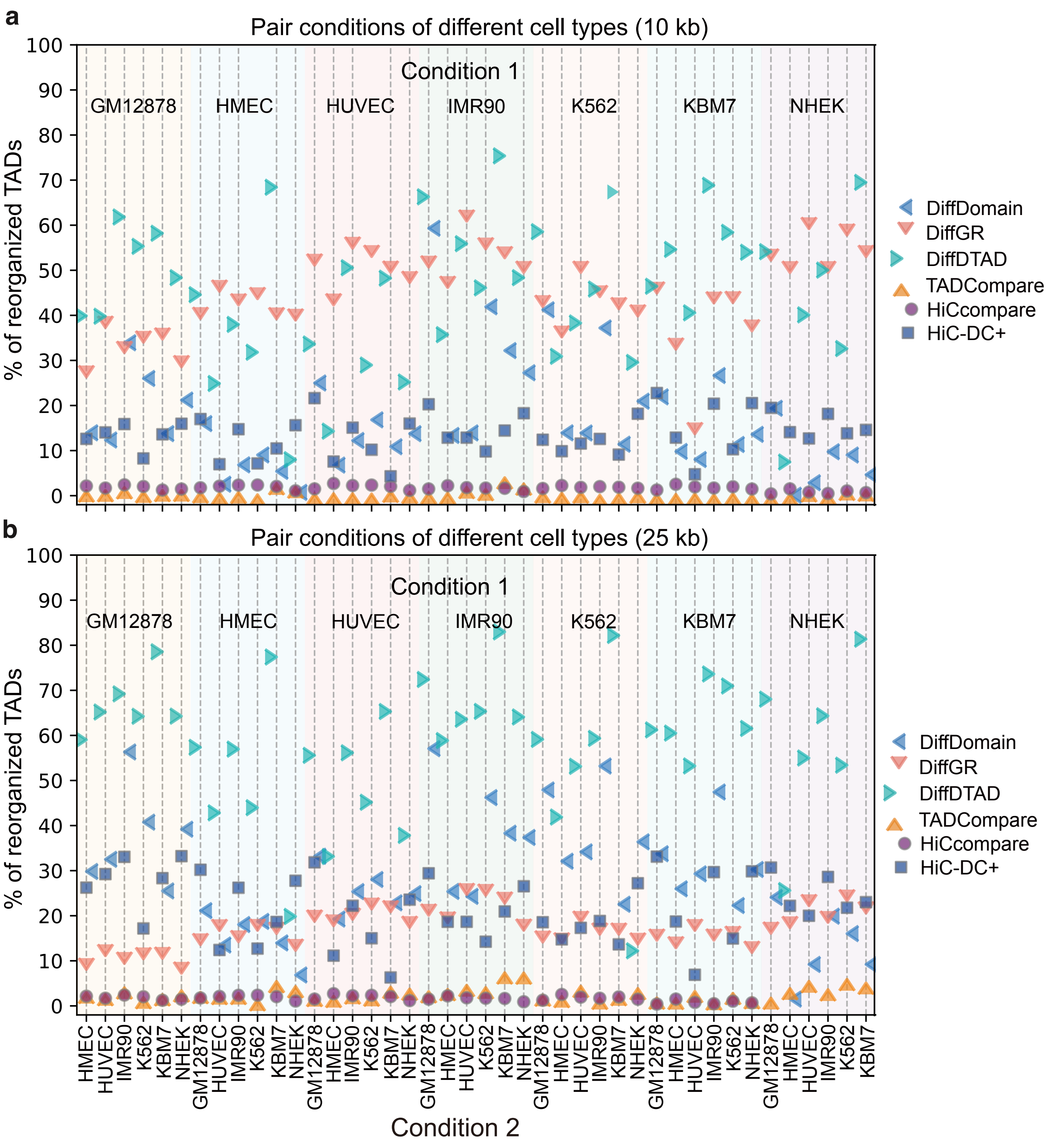

Supplementary Figure 7: Method comparison using the percentages of identified reorganized TADs between pairs of human cell lines. The resolution of Hi-C data are 10 kb **a** and 25 kb **b**. The proportions of reorganized TADs identified by HiCcompare and TADCompare are below 5%, suggesting that HiCcompare and TADCompare are too conservative. The proportions of reorganized TADs identified by HiC-DC+ are small (below 30%) while the FPRs of HiC-DC+ are high (ranging from 25% to 70% (Supplementary Fig. 6)), suggesting that HiC-DC+ is not optimal for identifying reorganized TADs. These results show that both HiC-DC+ and HiCcompare, specifically designed for identifying differential chromatin interactions, are not optimal for identifying reorganized TADs.

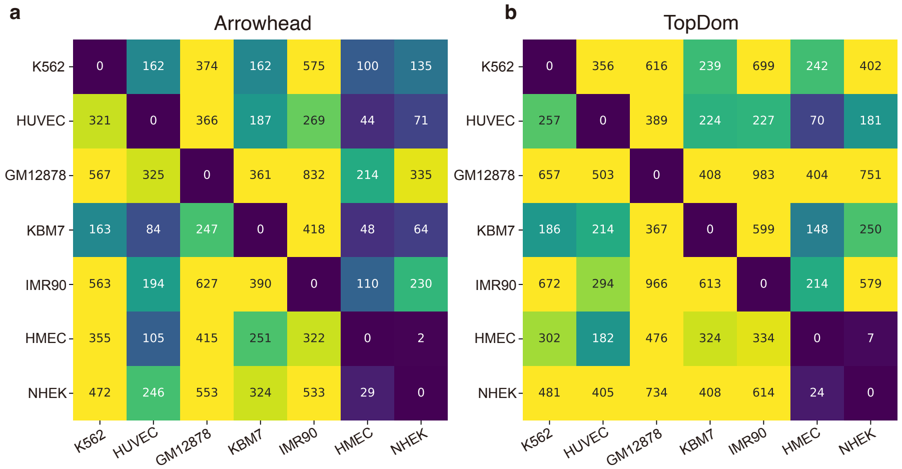

Supplementary Figure 8: Comparing DiffDomain with TADsplimer [4] in terms of the numbers of *split* and *merge* TADs. **a** TADs are called by Arrowhead, then the subset of reorganized TADs is identified by DiffDomain. **b** TADs are called by TopDom, then the subset of reorganized TADs is identified by DiffDomain. For each pair of cell types, the number of *split* and *merge* TADs is calculated as the summation of the number of *split* TADs and the number of *merge* TADs, the same as the calculation in Fig. 2e in Wang et al. [4]. Overall, the results in **b** are much more similar to the original results in Fig. 2e [4] than the results in **a**. This is expected because TopDom has similar performance with the built-in TAD caller in TADsplimer.

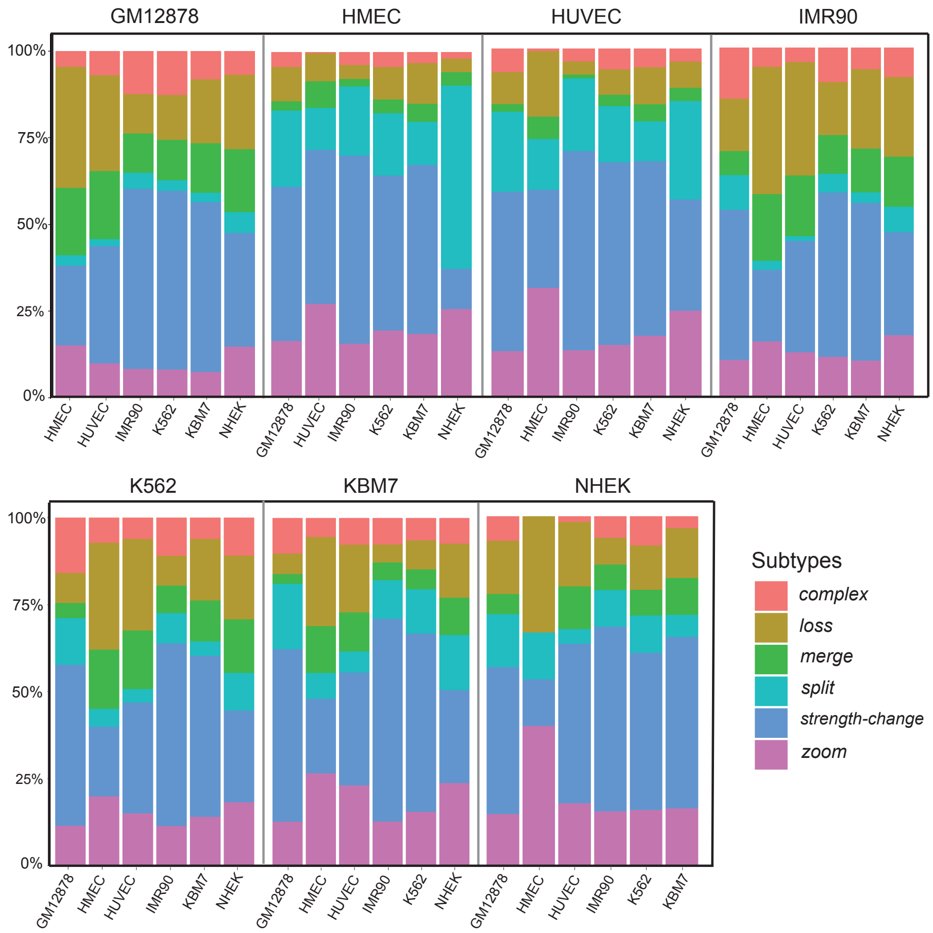

Supplementary Figure 9: Percentages of subtypes of reorganized TADs between human cell lines that are identified by DiffDomain. Cell lines on the top of stacked barplots are condition 1, cell lines on the X-axis are condition 2.

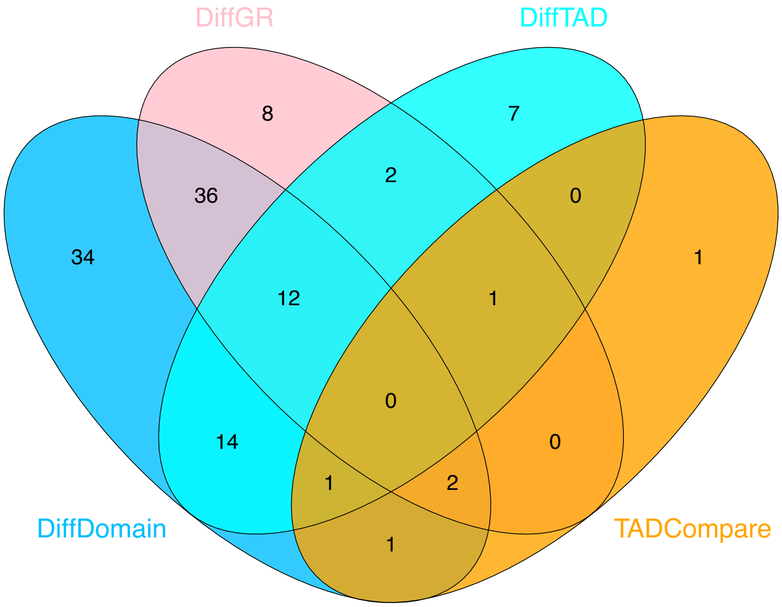

Supplementary Figure 10: Venn diagram showing the agreements between the lists of correctly identified truly reorganized TADs. There are 34 truly reorganized TADs are only correctly identified as such by DiffDomain. In contrast, only 8, 7, and 1 truly reorganized TADs are specifically correctly identified as such by DiffGR, DiffTAD, and TADCompare, respectively. Compared with DiffDomain, DiffGR, DiffTAD, and TADCompare only uniquely identify 11, 10, and 1 truly reorganized TADs, respectively, demonstrating the advantage of DiffDomain.

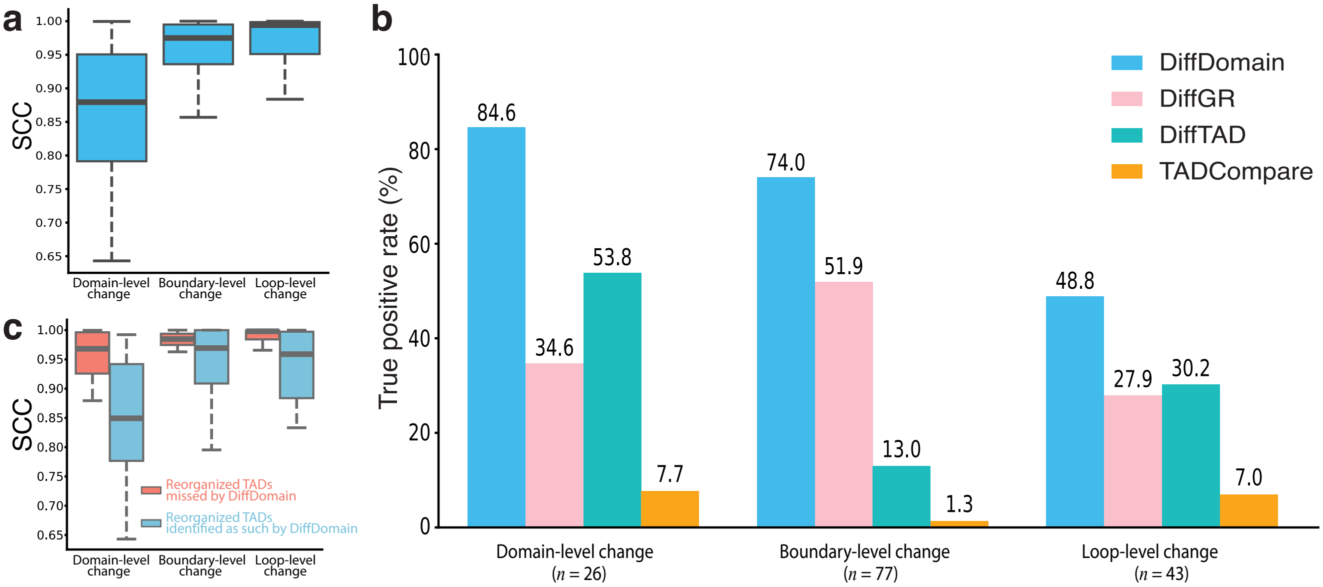

Supplementary Figure 11: Method comparison based on groups of truly reorganized TADs. **a** Boxplot showing the distribution of SCC score of truly reorganized TADs between 146 pairs of comparisons. Each data point represents the SCC score between a pair of Hi-C contact maps for a truly reorganized TAD. **b** Barplot showing TPRs of DiffDomain and alternative methods across the three groups of truly reorganized TADs. **c** Boxplot comparing the SCC scores between the correctly identified truly reorganized TADs and those missed by DiffDomain. The three groups of truly reorganized TADs, domain-level change, boundary-level change, and loop-level change, are broadly defined based on the original descriptions of changes in these TADs in the literature.

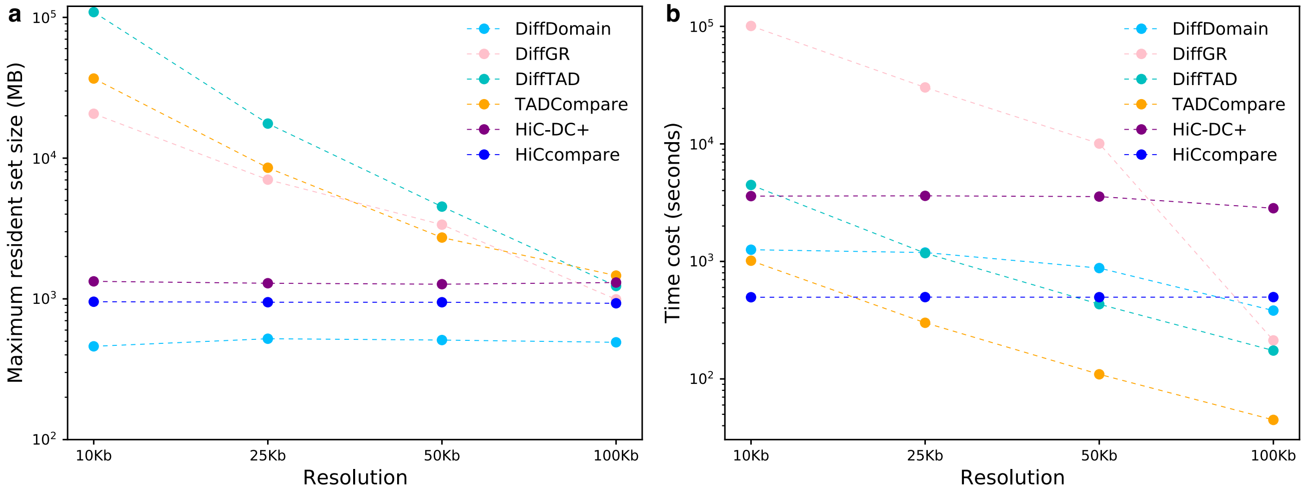

Supplementary Figure 12: Method comparison by memory usage and computation time. **a** Memory usage comparison in terms of maximum resident set size (MB). **b** Computation time comparison in terms of time cost (seconds). Statistics are estimated by Linux kernel function */usr/bin/time -f "%M %e"*. Each dot represents the average from 10 repeated experiments. Note that DiffDomain, HiC-DC+, and HiCcompare have stable memory usage and computation time because their bottleneck is extracting contact matrices from .hic files by *straw* package.

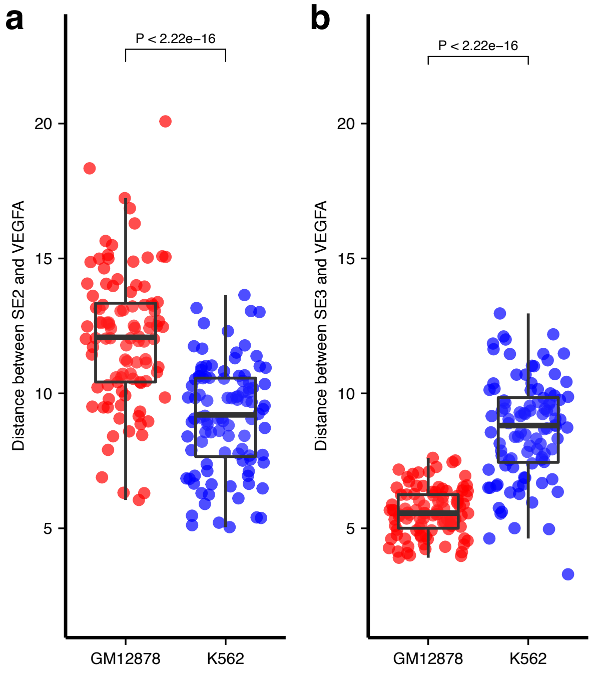

Supplementary Figure 13: Spatial proximities between VEGFA and super-enhancers in GM12878 and K562. **a** Distances between VEGFA and SE2 (super-enhancer Chr6:43871593-43959002). **b** Distances between VEGFA and SE3 (super-enhancer Chr6:44008142-44046149). Each dot represents a Euclidean distance between VEGFA and a super-enhancer in a given 3D structure. Totally, 100 3D structures for each cell type are generated by Chrom3D [41]. In the box plots, the middle line represents the median; the lower and upper lines correspond to the first and third quartiles; and the upper and lower whiskers extend to values no farther than 1.5×IQR. One-sided P value is computed by the Wilcoxon test.

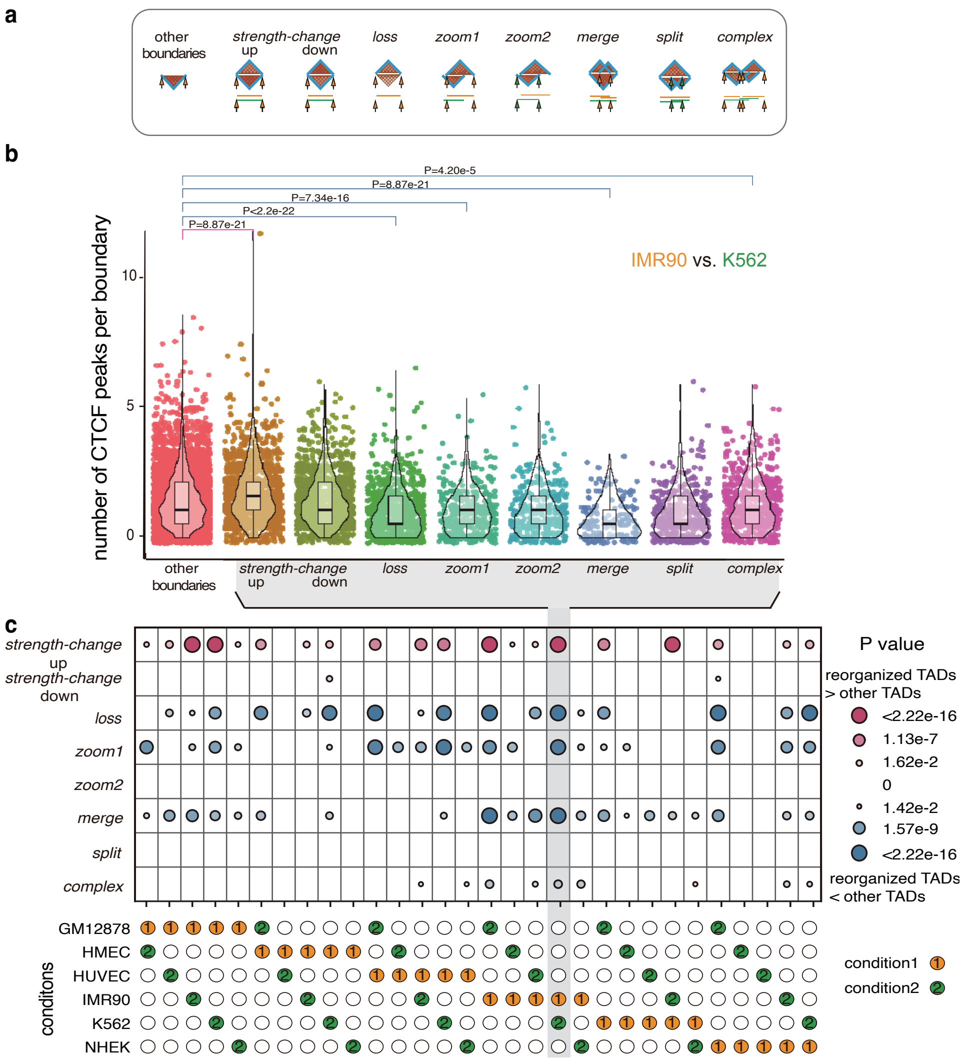

Supplementary Figure 14: Variations in the number of CTCF peaks related to reorganized TADs. **a** Cartoon diagram illustrating the comparison of CTCF peaks between different TAD boundary groups. The heatmap diagram illustrates TADs in condition 1 (top) and condition 2 (bottom), with lines representing TAD regions of the same condition sharing the same color. The first group are TAD boundaries from condition 2 TADs not overlapping with the subset of condition 1 TADs reorganized in condition 2, serving as a control. This group of TAD boundaries is called as other boundaries. Subsequent groups are defined by TAD reorganization subtypes. These include TAD boundaries from *strength-change up* TADs, *strength-change down* TADs, *loss* TADs, and zoom TADs (split into two groups: *zoom 1*, lost in condition 2; *zoom 2*, gained in condition 2). Addition groups include TAD boundaries lost due to TAD merging (*merge*) and TAD boundaries gained due to TAD splitting (*split*). Lastly, TAD boundaries associated with *complex* TADs but existing only in condition 1 are classified as *complex*. These groups are illustrated using vertical arrows. The number of CTCF peaks in each group are all counted using data from condition 2. **b** Boxplots showing the number of CTCF peaks across the TAD boundary groups. Here, condition 1 is IMR90, condition 2 is K562. Each dot represents one TAD boundary. The number of CTCF peaks in the group of other boundaries is compared with those from the remaining boundary groups. *P* values are computed using one-sided MannWhitney U test, a nonparametric test dealing with asymmetric distributions of the number of CTCF sites. For the sake of clarity, insignificant P values ($P > 0.05$) are excluded from the boxplot. **c** Heatmap showing the comparison of the number of CTCF peaks between the group of other boundaries and the remaining boundary groups across pairs of cell types. Each column corresponds to a specific cell type pair, with cell types indicated below the heatmap. Each row represents one TAD group. The heatmap cells represent significant levels ($\log_{10}(P)$) computed from comparing the corresponding TAD boundary group (row) with the group of other boundaries within a given cell type pair (column). Insignificant P values ($P > 0.05$) are left out from the heatmap to enhance clarity. Similar significant results are observed across the various cell type pairs. In the box plots, the middle line represents the median; the lower and upper lines correspond to the first and third quartiles; and the upper and lower whiskers extend to values no farther than 1.5×IQR.

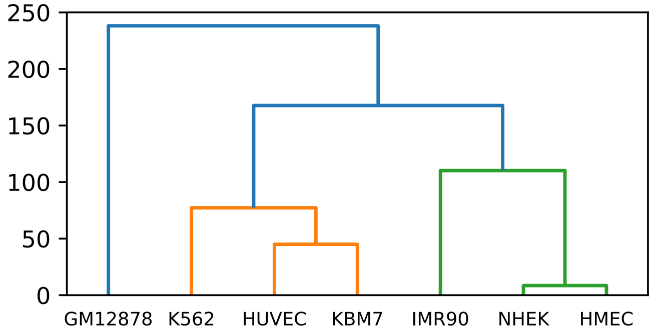

Supplementary Figure 15: Hierarchical clustering of cell types using the number of *split* TADs.

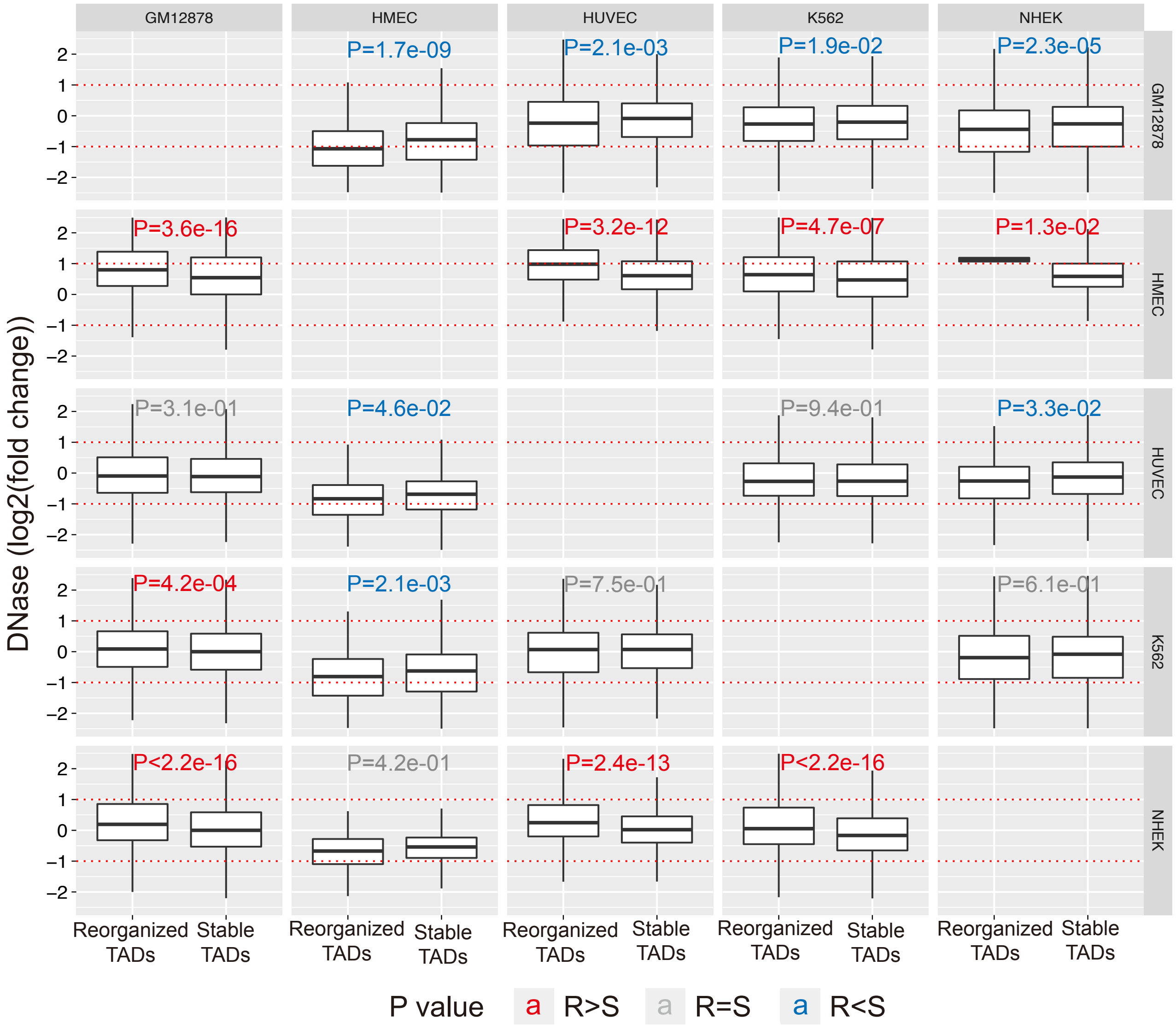

Supplementary Figure 16: Reorganized TADs tend to gain chromatin accessibility. We describe the analysis for the pair of GM12878 and HMEC cell types (first column and second row). we grouped GM12878 TADs into two groups: 1) TADs that are reorganized in HMEC cell type, called reorganized TADs; 2) the other TADs, called stable TADs. For each GM12878 TAD, we compute the fold-change of DNase peak coverage of the TAD in HMEC cell type to the coverage in GM12878 cell type. Then the fold-change is log_2 transformed, where a positive/negative value means that the TAD gains/losses chromatin accessibility in HMEC. The boxplots show that the reorganized TADs have significantly $(P = 3.6 \times 10^{-16})$ higher coverage of chromatin accessibility than the stable TADs. We repeat the analysis to the other pairs of cell types. Here each column represents the cell type where TADs are called, i.e., condition 1. Each row represents the cell type where TADs are tested whether they are reorganized, i.e., condition 2. The P values (P) in red and blue colors mean that reorganized TADs have significantly $(P \leq 0.05)$ higher and lower mean change in chromatin accessibility. While the p values in grey mean that the two groups of TADs have no significant difference in change of chromatin accessibility. P values are computed by Mann-Whitney test. IMR90 and KBM7 are excluded because they do not have the ENCODE DNase data at the UCSC Genome Browser. Although reorganized TADs have significantly lower fold-change in chromatin accessibility in some pairs of comparisons, the levels of significance are much smaller than those from the comparisons where reorganized TADs have significantly higher fold-change in chromatin accessibility. In the box plots, the middle line represents the median; the lower and upper lines correspond to the first and third quartiles; and the upper and lower whiskers extend to values no farther than 1.5×IQR.

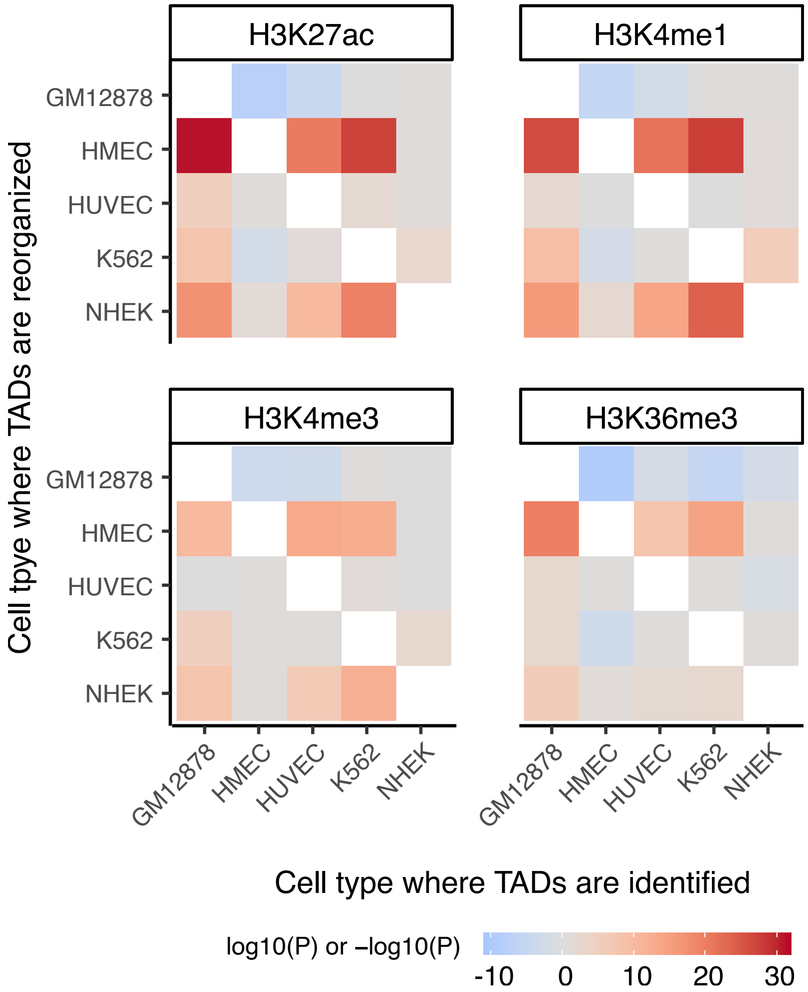

Supplementary Figure 17: Reorganized TADs tend to gain histone modifications that are signals for enhancers and active transcription. P values are calculated as the same in the case of DNase signals shown in Supplementary Fig. 16. *P* values are transformed by $-\log_{10}(P)$ if reorganized TADs have higher fold-change in signals than the other TADs. Otherwise, *P* values are transformed by $\log_{10}(P)$. Missing off-diagonal elements mean that the corresponding marks do not exist in the corresponding cell types.

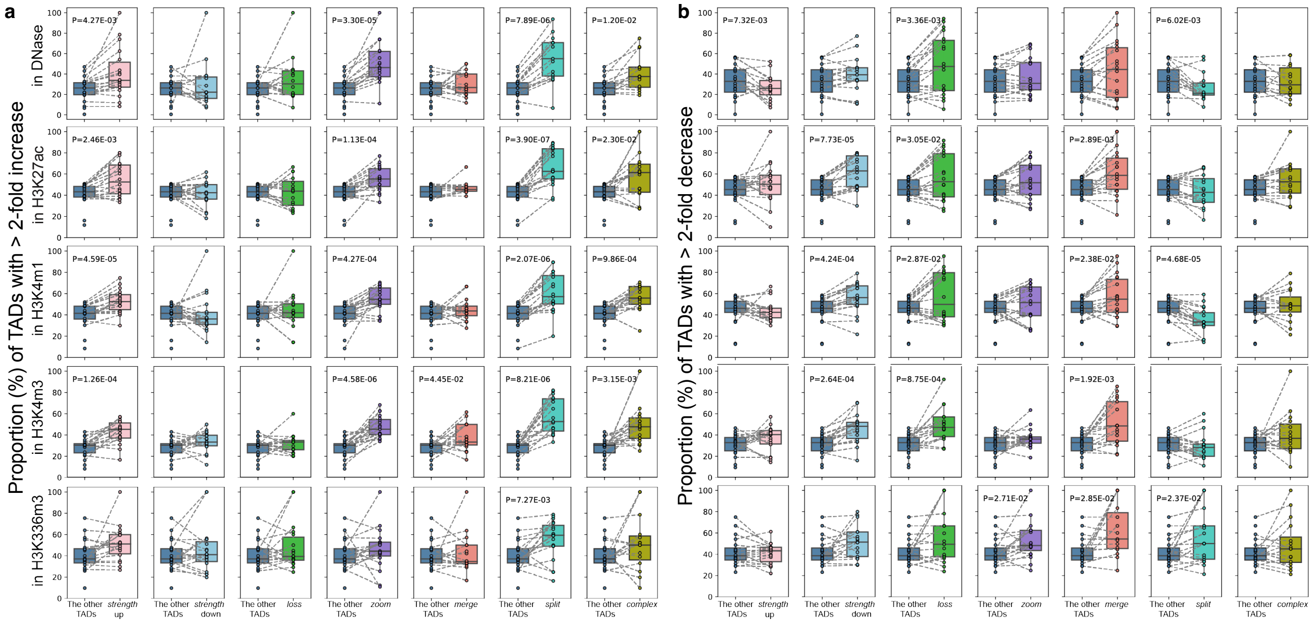

Supplementary Figure 18: Distinct associations between TAD reorganization subtypes and chromatin accessibility as well as histone modifications. **a** Boxplot comparing reorganized TADs with subtypes of reorganized TADs in terms of increasing in chromatin accessibility and histone modification signals. We explain the boxplot for DNase peak coverage (*top left*). The proportion (Y-axis) is computed as follows: within a given cell type pair, for the subset of *strength-change up* TADs, the proportion is computed as M/N. Here, M is the number of *strength-change up* TADs with at least 2-fold increase in DNase peak coverage, and N is the number of *strength-change up* TADs with increase (fold-change > 1) in DNase peak coverage. Similarly, the proportion is computed for the other TAD subsets. The proportion represents the fraction of TADs with at least 2-fold increasing in DNase peak coverage among the TADs with increased DNase peak coverage, The connection between two proportions from the same cell type pair comparison is indicated by a dash line. In total, the dataset contains 20 pairs of data points, derived from 20 pairwise comparisons among GM12878, HMEC, HUVEC, K562, and NHEK cell types. Insignificant *P* values are omitted from the boxplots for clarity. One-sided P value is computed by the Wilcoxon test. **b** Boxplot comparing reorganized TADs with subtypes of reorganized TADs in terms of decreasing in chromatin accessibility and histone modification signals.

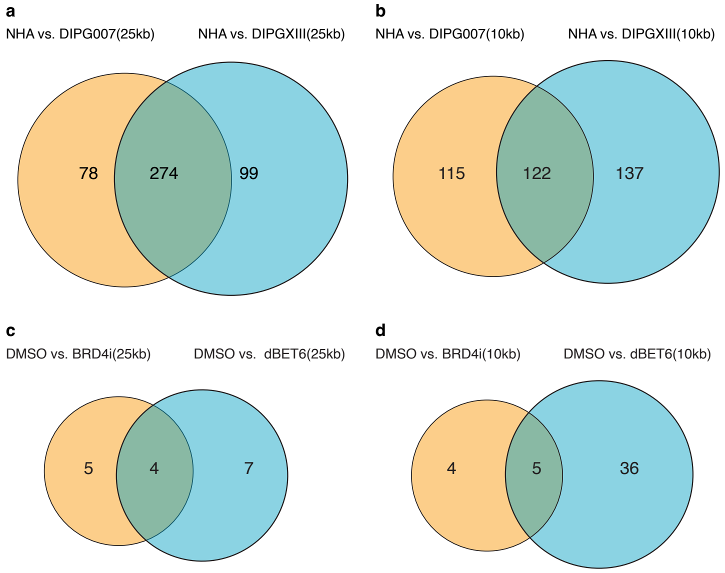

Supplementary Figure 19: Agreements between reorganized TADs in DIPG cell lines. **a** Venn diagram showing the agreement between (1) NHA TADs that are reorganized in DIPG007 and (2) NHA TADs that are reorganized in DIPGXIII. Hi-C resolution is 25 kb. **b** Same as **a**, except for Hi-C resolution at 10 kb. **c** Venn diagram showing the agreement between (1) DMSO TADs that are reorganized in BRD4i and (2) DMSO TADs that are reorganized in dBET6. Hi-C resolution is 25 kb. **d** Same as **c**, except for Hi-C resolution at 10 kb. Abbreviation: NHA, normal human astrocytes; DIPG007 and DIPGXIII, pediatric high-grade glioma patient-derived cell lines; DMSO, DIPG007 with dimethyl sulfoxide treatment (used as control treatment); BRD4i, DIPG007 with the BET BRD inhibitor treatment; dBET6, DIPG007 with BRD degrader treatment.

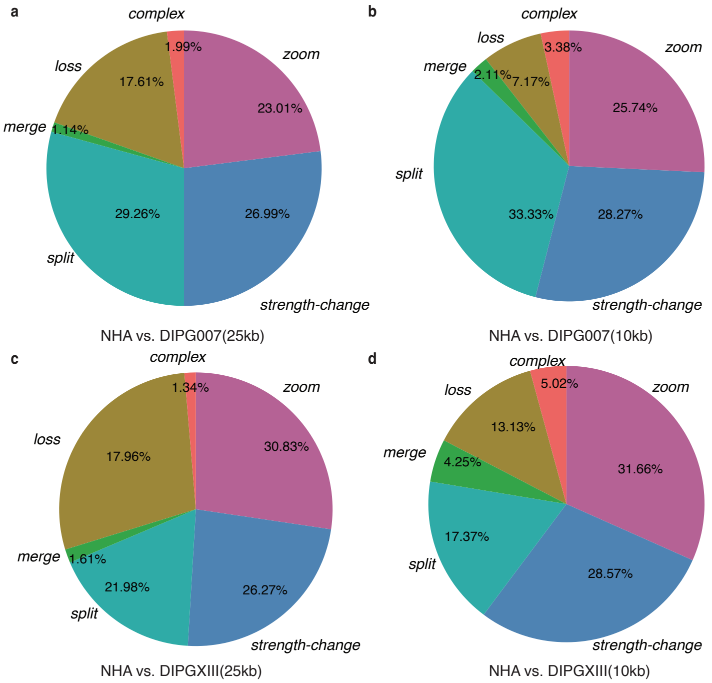

Supplementary Figure 20: Percentages of subtypes of NHA TADs that are reorganized in DIPG cell lines. **a** Pie chart showing the percentages of subtypes of NHA TADs that are reorganized in DIPG007. Hi-C resolution is 25 kb. **b** Same as **a**, except for Hi-C resolution at 10 kb. **c** Pie chart showing the percentages of subtypes of NHA TADs that are reorganized in DIPGXIII. Hi-C resolution is 25 kb. **d** Same as **c**, except for Hi-C resolution at 10 kb. Abbreviation: NHA, normal human astrocytes; DIPG007 and DIPGXIII, pediatric high-grade glioma patient-derived cell lines.

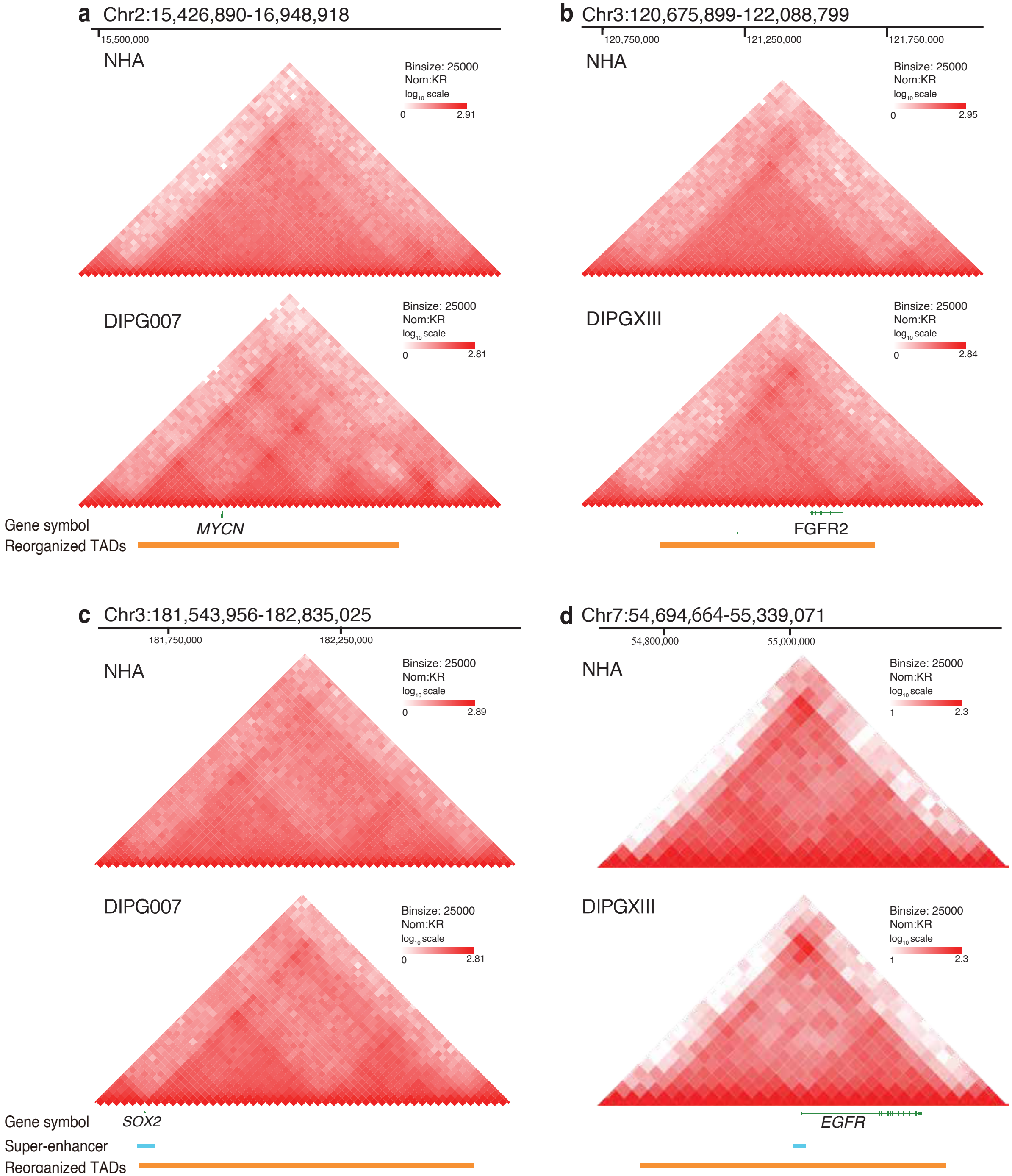

Supplementary Figure 21: Examples of reorganized TADs in DIPG cell lines that harbor oncogenes and super-enhancers. **a** A NHA TAD reorganized in DIPGXIII. The reorganized TAD harbors oncogene *MYCN*. **b** A NHA TAD reorganized in DIPG007. The reorganized TAD harbors oncogene *FGFR2*. **c** A NHA TAD reorganized in DIPGXIII. The reorganized TAD harbors both oncogene *SOX2* and a DIPGXIII sup-enhancer. **d** A NHA TAD reorganized in DIPG007. The reorganized TAD harbors oncogene *EGFR*. The upstream of the reorganized TAD contains a DIPG007 super-enhancer. Here, a super-enhancer is associated with a reorganized TAD if their 1D genomic distance is within 50 kb.

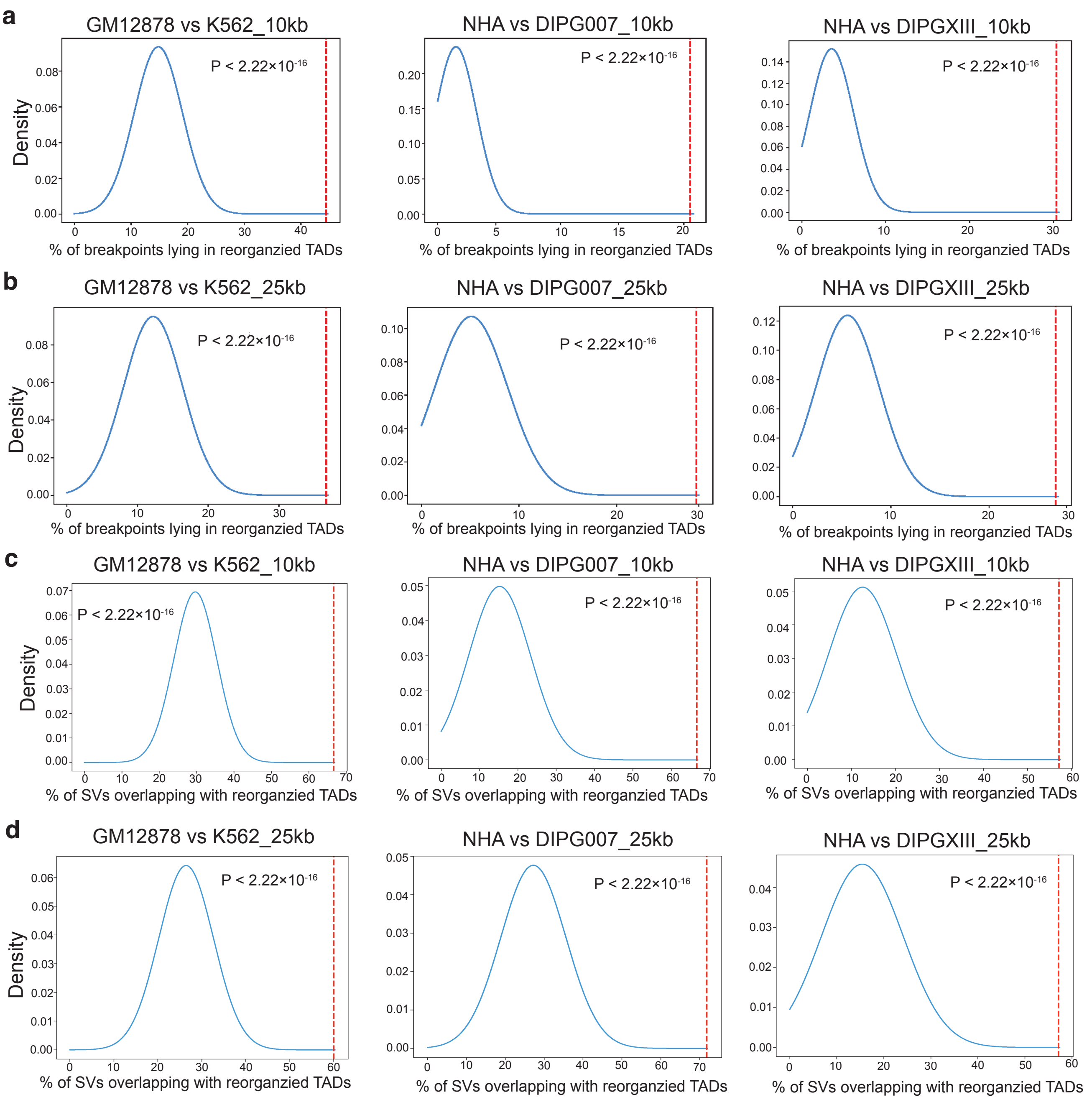

Supplementary Figure 22: Higher proportions of SVs with reorganized TADs than random cases. **a,b** Density curves comparing the proportions (vertical dashed lines) of breakpoints lying in reorganized TADs with the proportions (blue curves) from random cases. The reorganized TADs are GM12878 TADs reorganized in K562, NHA TADs reorganized in DIPG007, and NHA TADs reorganized in DIGPXIII, respectively. Hi-C resolution is chosen at 10 kb **a** and 25 kb **b**. **c,d** Density curves comparing the proportions (vertical dashed lines) of the genomic regions of SVs that overlap with reorganized TADs with the proportions (blue curves) from random cases. Hi-C resolution is chosen at 10 kb **c** and 25 kb **d**. To calculate statistical significance, we randomly sample the same number of reorganized TADs and calculate the statistics. The sampling procedure is repeated 10,000 times and empirical P value is calculated. SVs with their genomic regions overlappping with reorganized TADs can result from either having their breakpoints lying in a reorganized TAD or containing one or more reorganized TADs (Fig. 4a). Thus, we stratified the simulation based on the two types of associations between SVs and reorganized TADs.

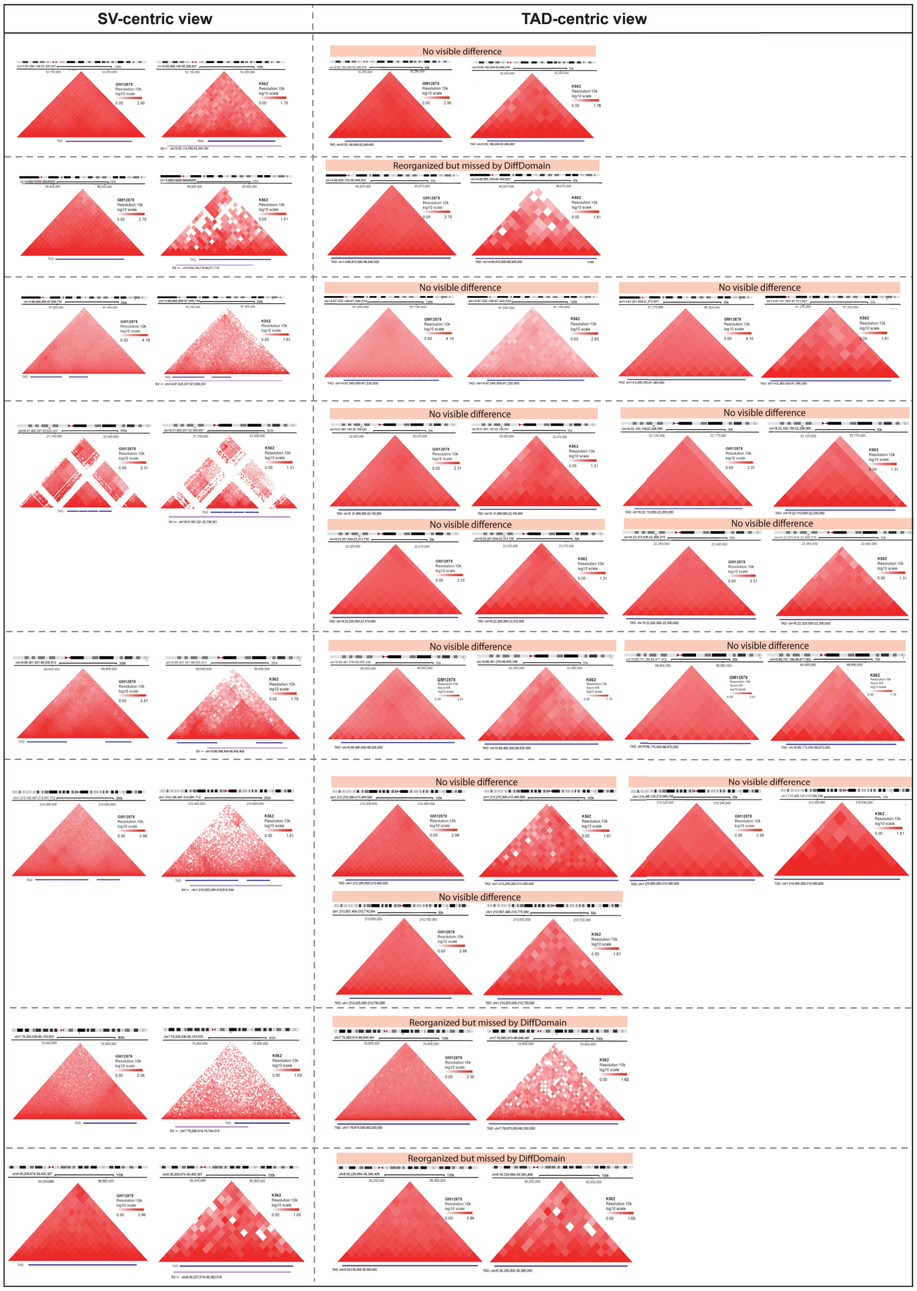

Supplementary Figure 23: Visual examination on K562 SVs without reorganized TADs. The first two columns are SV-centric visualization, showing Hi-C contact maps from both conditions covering a region containing a specific SV and its associated TADs. First track below the heatmap represents TAD regions associated with a specific SV. Second track represents the specific SV region. Subsequent columns are TAD-centric visualization, showing a zoomed-in view of Hi-C contact maps for each of the TAD region associated with the SV region. Here, only the track representing a TAD region is shown below the heatmaps. The label “no visible difference” represents a manually draw conclusion that the TAD associated with the SV has no clear visual distinction between its two Hi-C contact maps from the two conditions, confirming DiffDomain’s results. The label “reorganized but missed by DiffDomain” represents that a manual assessment identifies visual difference between conditions for the TAD associated with the SV, although DiffDomain did not detect them. Note that, the visualization exclusively visualize SVs lacking reorganized TADs based on DiffDomain results. Among these SVs, 3 SVs have reorganized TADs but are not detected by DiffDomain. The remaining 5 SVs may lack associated TAD reorganization. The visualization is conducted using the Nucleome Browser. Abbreviations: ‘+-’, deletion;‘-+’, 5′ to 3′ fusion; ‘--’, 5′ to 5′ fusion; ‘++’, 3′ to 3′ fusion.

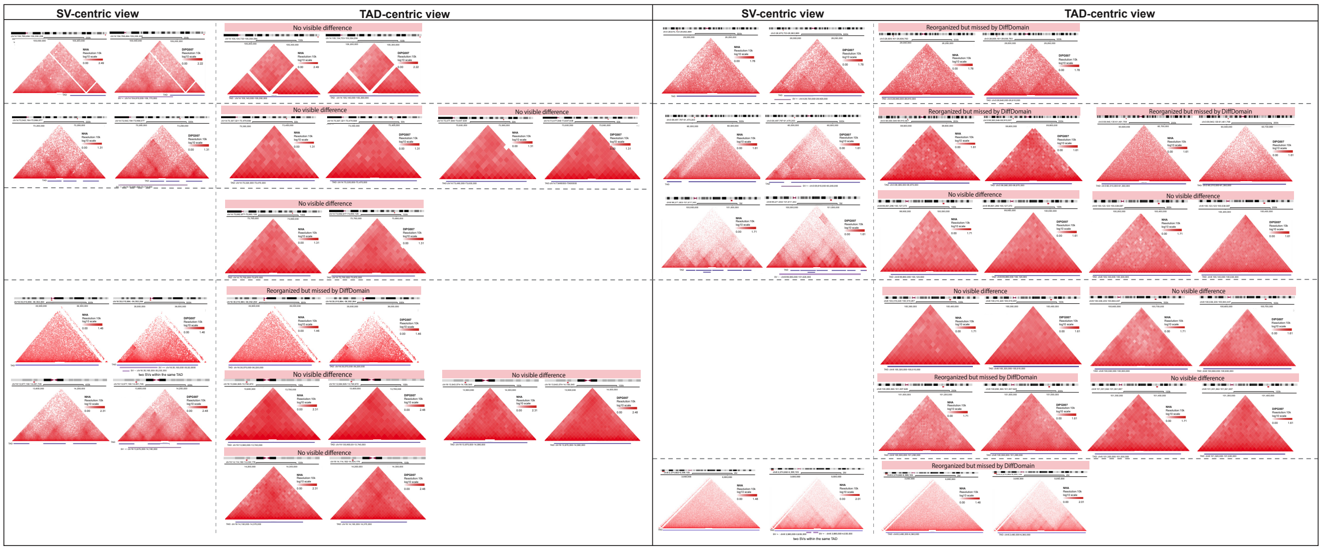

Supplementary Figure 24: Visual examination on DIPG007 SVs without reorganized TADs. The layout scheme is the same to Supplementary Fig. 23. Note that, the visualization exclusively visualize SVs lacking reorganized TADs, as determined by DiffDomain's results. Among these SVs, 7 DIPG007 SVs have reorganized TADs but are not detected by DiffDomain. The remaining 3 DIPG007 SVs may lack associated TAD reorganization.

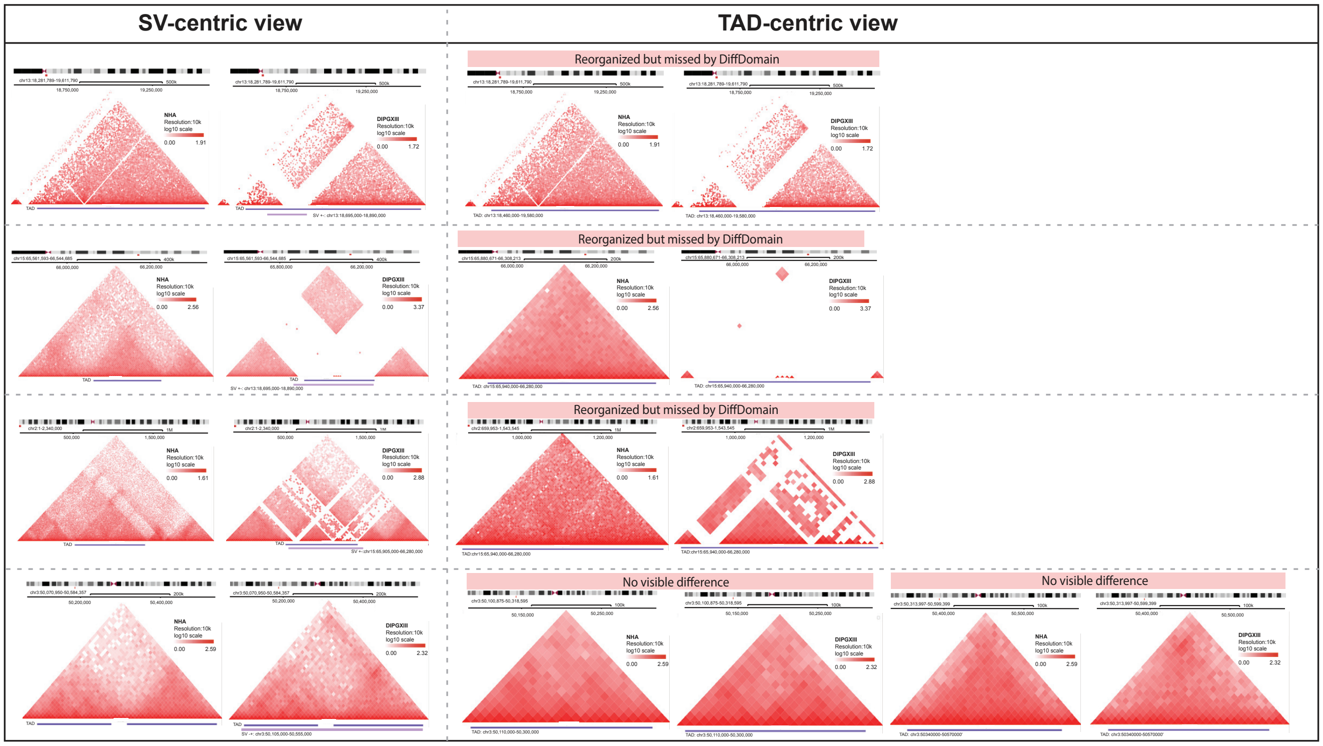

Supplementary Figure 25: Visual examination on DIPGXIII SVs without reorganized TADs. The layout scheme is the same to Supplementary Fig. 23. Note that, the visualization exclusively visualize SVs lacking reorganized TADs, as determined by DiffDomain's results. Among these SVs, 3 DIPG007 SVs have reorganized TADs but are not detected by DiffDomain. The remaining 1 DIPG007 SV may lack associated TAD reorganization.

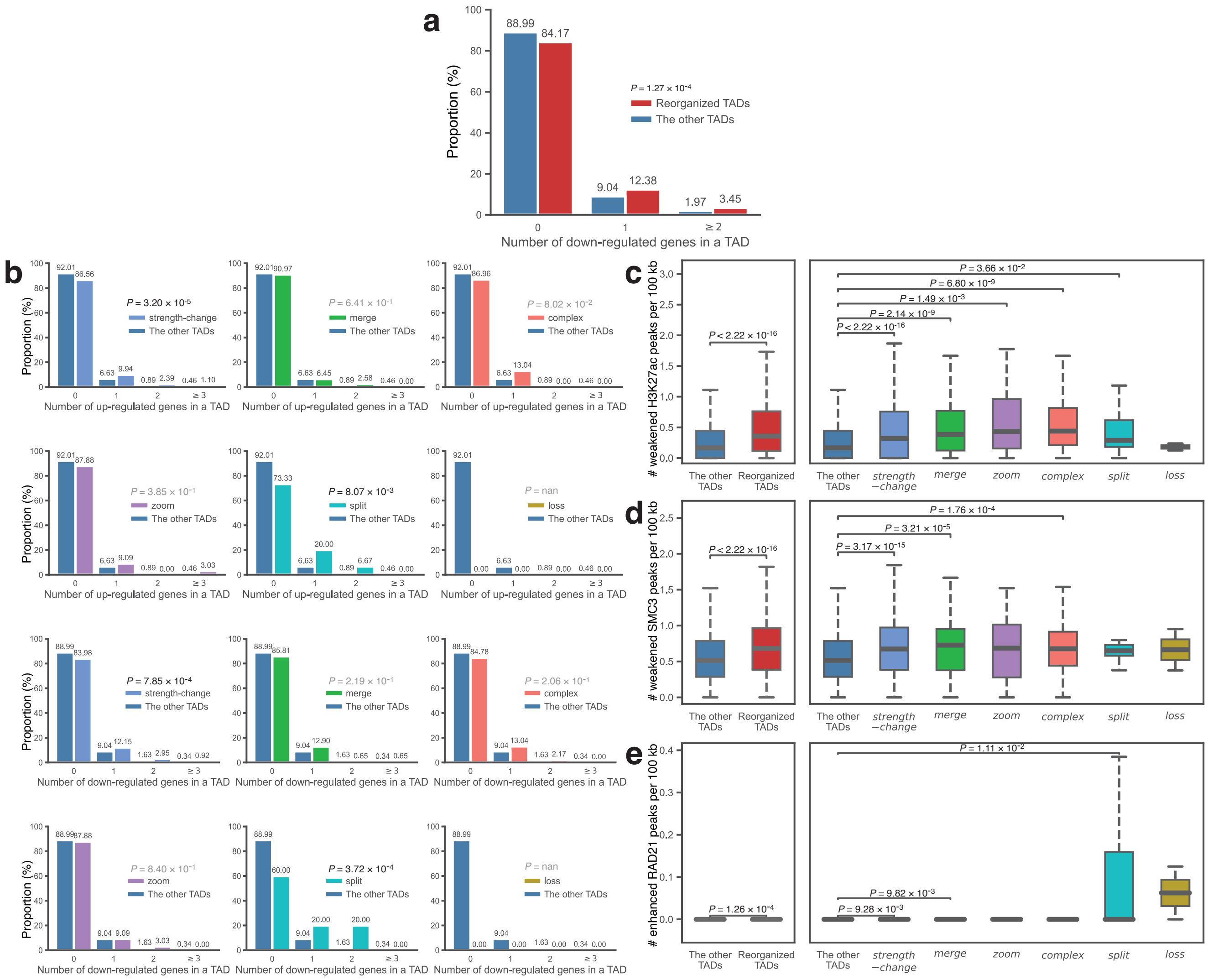

Supplementary Figure 26: Reorganized TADs after SARS-CoV-2 infection are associated with higher numbers of down-regulated genes and weakened peaks of H3K27ac, SMC3, and RAD21. **a** Barplot comparing the number of down-regulated genes in the reorganized TADs and the other TADs. **b** Barplots comparing the numbers of up- and down-regulated genes between the subtype of reorganized TADs and the other TADs. Boxplots comparing the numbers of weakened H3K27ac peaks **c**, weakened SMC3 peaks **d**, and enhanced RAD21 peaks **e** per 100 kb. *Left*: comparing reorganized TADs with the other TADs; *right*: comparison stratified by the subtypes of reorganized TADs. Note that the number of enhanced RAD21 peaks called by MAnorm2 is small, thus, the values in **e** are close to 0. TADs are called in mock-infected A549-ACE2 cells, reorganized TADs are called in SARS-CoV-2 infected A549-ACE2 cells. Differentially expressed genes are called by DESeq2 [42]. Differential peaks are called by MAnorm2 [43]. Boxplots are drawn by the *seaborn.boxplot* function in Python 3. In the box plots, the middle line represents the median; the lower and upper lines correspond to the first and third quartiles; and the upper and lower whiskers extend to values no farther than 1.5×IQR. Data is downloaded from Wang et al. [10].

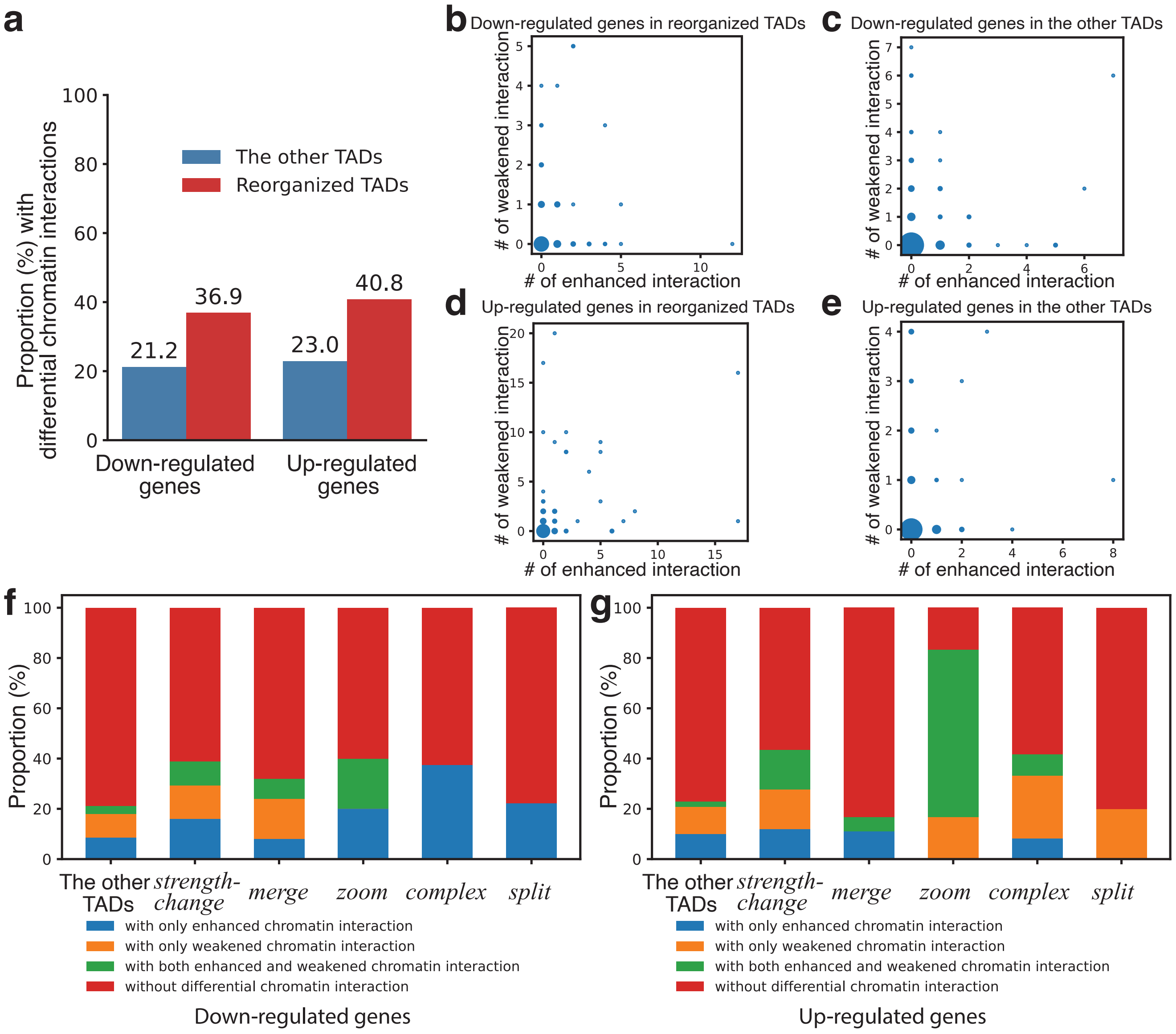

Supplementary Figure 27: Association between differentially expressed genes and differential chromatin interactions in reorganized TADs after SARS-CoV-2 infection. **a** Barplot showing the proportions of down-regulated genes and up-regulated genes that have at least one differential chromatin interactions. The genes are further categorized into two groups: those located in reorganized TADs and those located in the other TADs. **b** Scatter plot showing the number of enhanced chromatin interactions (X-axis) against the number of weakened chromatin interactions (Y-axis) for down-regulated genes locating in reorganized TADs. Point size proportions to the number of genes. **c** Similar to **b** except that the down-regulated genes locate in the other TADs. **d** Scatter plot showing the number of enhanced chromatin interactions and the number of weakened chromatin interactions for up-regulated genes located in reorganized TADs. **e** Similar to **d** except that the up-regulated genes locate in the other TADs. **f** Stacked barplot showing the proportion of down-regulated genes with different combinations of differential chromatin interactions: with only enhanced chromatin interaction, with only weakened chromatin interaction, with both enhanced and weakened chromatin interaction, and without differential chromatin interaction. The X-axis represents the other TADs and the subtypes of reorganized TADs that harbor the down-regulatead genes. **g** Similar to **f**, except for up-regulated genes.

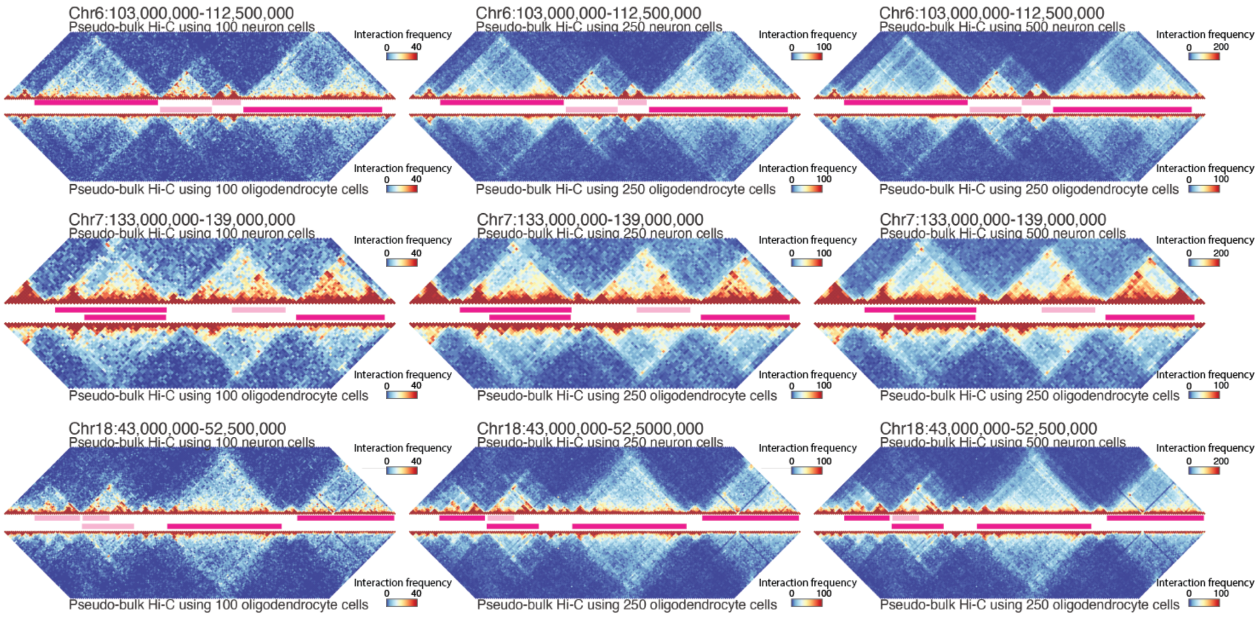

Supplementary Figure 28: Examples of DiffDomain application to single-cell chromatin contact maps. Visualization of merged Hi-C contact maps and identified reorganized TADs (dark pink horizontal lines) in genomic regions on Chromosome 6 (*Top*), Chromosome 18 (*Middle*), and Chromosome 7 (*Bottom*). The pair of conditions are neurons and oligodendrocytes. TADs are called in neurons. The numbers of sampled cells are (100, 100), (250, 250), (500, 250), respectively.

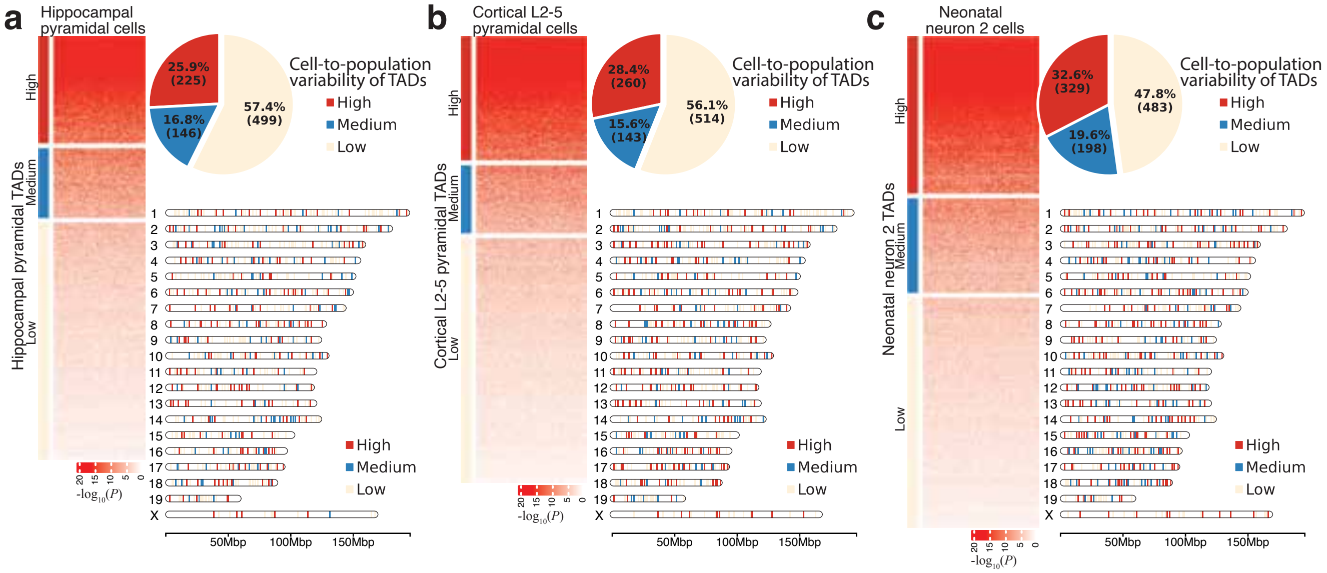

Supplementary Figure 29: Characterization of differential cell-to-population variability of TADs in three cell types. **a** Characterization in hippocampal pyramidal cells. *Left*: Heatmap showing high, median, and low cell-to-population variational TADs. Columns represent individual hippocampal pyramidal cells. Rows represent individual TADs. Values in the heatmap represent the -log$_{10}(P)$. P value is computed by DiffDomain when comparing scHi-C contact map of a TAD (row) in an individual cell (column) to the pseudo-bulk Hi-C contact map that are created using all scHi-C data from the cell type. Classification of TADs is done by hierarchical clustering. *Top right*: Pie chart showing the percentages of the high, median, and low cell-to-population variational TADs. *Bottom right*: Chromosome map showing the genomic locations of high, median, and low cell-to-population variational TADs. **b** Characterization in cortical L2-5 pyramidal cells. **c** Characterization in neonatal neuron 2 cells. These analyses are the repetition of the cell-to-population analysis neonatal neuron 1 cell type (Fig. 7a,b,d) to these three cell types.

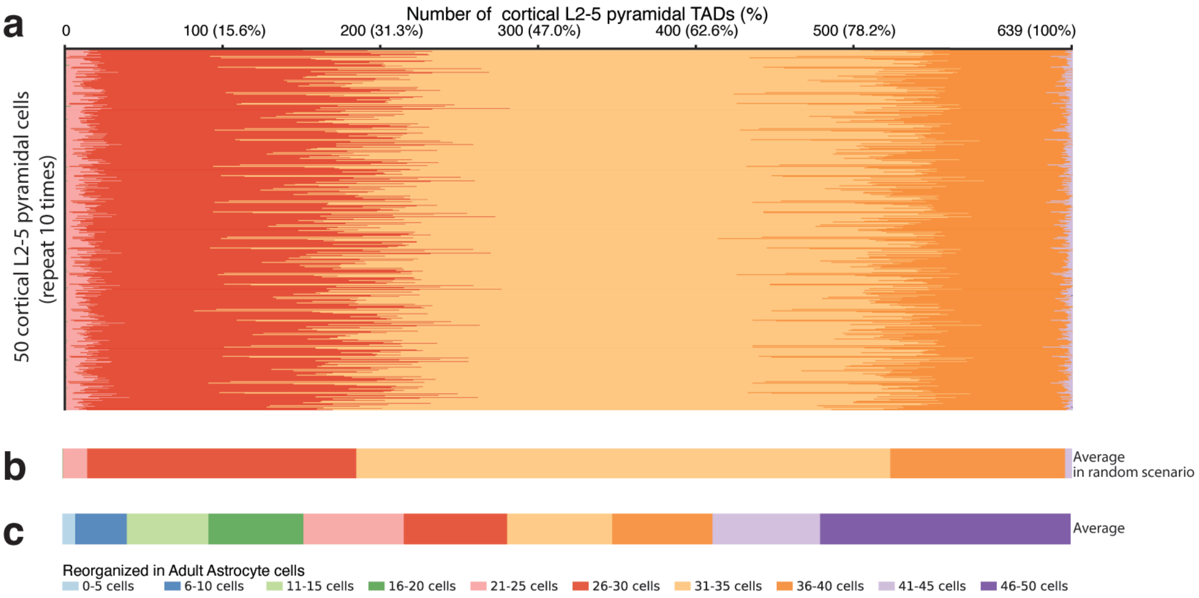

Supplementary Figure 30: Cell-to-cell variability in random scenarios. **a** Stacked barplots showing the number (the percentage) of the cortical L2-5 pyramidal TADs that are reorganized in a varied number (0 to 50) of adult astrocytes in random scenarios. The layout is the same as Fig. 7f. In the simulation, equal-numbered reorganized TADs are randomly assigned in pairwise comparisons. Rows represent 50 cortical L2-5 pyramidal cells with 10 times repetition per cell. **b** Stacked barplot representing the average number (the percentage) in the simulation in **a**. **c** Stacked bar graph representing the average number (the percentage) in real cases (also shown in Fig. 7f). This figure clearly shows that cortical L2-5 pyramidal TADs are mostly reorganized in 26-40 cells in random scenarios **a,b**, which are much different from the patterns in real case **c**.

Supplementary References

[1] Cresswell, K. G. & Dozmorov, M. G. TADCompare: an R package for differential and temporal analysis of topologically associated domains. *Front. Genet.* **11**, 158 (2020).

[2] Liu, H. & Ma, W. DiffGR: Detecting differentially interacting genomic regions from Hi-C contact maps. Preprint at *bioRxiv* https://doi.org/10.1101/2020.08.29.273698 (2020).

[3] Zaborowski, R. & Wilczynski, B. DiffTAD: Detecting differential contact frequency in topologically associating domains Hi-C experiments between conditions Preprint at *bioRxiv* https://doi.org/10.1101/093625 (2016).

[4] Wang, G. et al. TADsplimer reveals splits and mergers of topologically associating domains for epigenetic regulation of transcription. *Genome Biol.* **21**, 84 (2020).

[5] Stansfield, J. C., Cresswell, K. G., Vladimirov, V. I. & Dozmorov, M. G. HiCcompare: an R-package for joint normalization and comparison of Hi-C datasets. *BMC Bioinformatics* **19**, 279 (2018).

[6] Sahin, M. et al. HiC-DC+ enables systematic 3D interaction calls and differential analysis for Hi-C and HiChIP. *Nat. Commun.* **12**, 3366 (2021).

[7] Rao, S. S. et al. A 3D map of the human genome at kilobase resolution reveals principles of chromatin looping. *Cell* **159**, 1665–1680 (2014).

[8] Xiong, K. & Ma, J. Revealing Hi-C subcompartments by imputing inter-chromosomal chromatin interactions. *Nat. Commun.* **10**, 5069 (2019).

[9] Wang, J. et al. Epigenomic landscape and 3D genome structure in pediatric high-grade glioma. *Sci. Adv.* **7**, eabg4126 (2021).

[10] Wang, R. et al. SARS-CoV-2 restructures host chromatin architecture. *Nat. Microbiol.* **8**, 679–694 (2023).

[11] Jiang, Y. *et al.* The methyltransferase SETDB1 regulates a large neuron-specific topological chromatin domain. *Nat. Genet.* **49**, 1239–1250 (2017).

[12] Sofueva, S. et al. Cohesin-mediated interactions organize chromosomal domain architecture. *EMBO J.* **32**, 3119–3129 (2013).

[13] Tan, L. et al. Changes in genome architecture and transcriptional dynamics progress independently of sensory experience during post-natal brain development. *Cell* **184**, 741–758 (2021).

[14] The ENCODE Project Consortium. An integrated encyclopedia of DNA elements in the human genome. *Nature* **489**, 57–74 (2012).

[15] Wang, Y. et al. SEdb 2.0: a comprehensive super-enhancer database of human and mouse. *Nucleic Acids Res.* **51**, D280–D290 (2023).

[16] Whyte, W. A. et al. Master transcription factors and mediator establish super-enhancers at key cell identity genes. *Cell* **153**, 307–319 (2013).

[17] Chakravarty, D. et al. Oncokb: a precision oncology knowledge base. *JCO Precis. Oncol.* **1**, 1–16 (2017).

[18] Zufferey, M., Tavernari, D., Oricchio, E. & Ciriello, G. Comparison of computational methods for the identification of topologically associating domains. *Genome Biol.* **19**, 1–18 (2018).

[19] Zhu, X. et al. Nucleome Browser: an integrative and multimodal data navigation platform for 4D Nucleome. *Nat. Methods* **19**, 911–913 (2022).

[20] Li, A. et al. Decoding topologically associating domains with ultra-low resolution Hi-C data by graph structural entropy. *Nat. Commun.* **9**, 3265 (2018).

[21] Dixon, J. R. et al. Topological domains in mammalian genomes identified by analysis of chromatin interactions. *Nature* **485**, 376–380 (2012).

[22] Safran, M. et al. GeneCards Version 3: the human gene integrator. *Database* **2010**, baq020 (2010).

[23] Barski, A. *et al.* High-resolution profiling of histone methylations in the human genome. *Cell* **129**, 823–837 (2007).

[24] Heintzman, N. D. et al. Distinct and predictive chromatin signatures of transcriptional promoters and enhancers in the human genome. *Nat. Genet.* **39**, 311–318 (2007).

[25] Heintzman, N. D. et al. Histone modifications at human enhancers reflect global cell-type-specific gene expression. *Nature* **459**, 108–112 (2009).

[26] Yang, H. et al. A map of cis-regulatory elements and 3D genome structures in zebrafish. *Nature* **588**, 337–343 (2020).

[27] Goodman, J. V. et al. The chromatin remodeling enzyme CHD4 regulates genome architecture in the mouse brain. *Nat. Commun.* **11**, 3419 (2020).

[28] Huang, H. et al. CTCF mediates dosage-and sequence-context-dependent transcriptional insulation by forming local chromatin domains. *Nat. Genet.* **53**, 1064–1074 (2021).

[29] Szabo, Q. et al. Regulation of single-cell genome organization into TADs and chromatin nanodomains. *Nat. Genet.* **52**, 1151–1157 (2020).

[30] Lee, D.-I. & Roy, S. GRiNCH: simultaneous smoothing and detection of topological units of genome organization from sparse chromatin contact count matrices with matrix factorization. *Genome Biol.* **22**, 164 (2021).

[31] Bonev, B. et al. Multiscale 3D genome rewiring during mouse neural development. *Cell* **171**, 557–572 (2017).

[32] Winick-Ng, W. et al. Cell-type specialization is encoded by specific chromatin topologies. *Nature* **599**, 684–691 (2021).

[33] Xiao, T., Li, X. & Felsenfeld, G. The Myc-associated zinc finger protein (MAZ) works together with CTCF to control cohesin positioning and genome organization. *Proc. Nat. Acad. Sci. USA* **118**, e2023127118 (2021).

[34] Zhang, D. et al. Alteration of genome folding via contact domain boundary insertion. *Nat. Genet.* **52**, 1076–1087 (2020).

[35] Bertero, A. et al. Dynamics of genome reorganization during human cardiogenesis reveal an RBM20-dependent splicing factory. *Nat. Commun.* **10**, 1538 (2019).

[36] Xing, H., Wu, Y., Zhang, M. Q. & Chen, Y. Deciphering hierarchical organization of topologically associated domains through change-point testing. *BMC Bioinformatics* **22**, 183 (2021).

[37] Dixon, J. R. et al. Chromatin architecture reorganization during stem cell differentiation. *Nature* **518**, 331–316 (2015).

[38] Zhang, Y. et al. Transcriptionally active HERV-H retrotransposons demarcate topologically associating domains in human pluripotent stem cells. *Nat. Genet.* **51**, 1380–1388 (2019).

[39] Rowley, M. J. et al. Evolutionarily conserved principles predict 3D chromatin organization. *Mol. Cell* **67**, 837–852 (2017).

[40] Wang, X., Luan, Y. & Yue, F. EagleC: A deep-learning framework for detecting a full range of structural variations from bulk and single-cell contact maps. *Sci. Adv.* **8**, eabn9215 (2022).

[41] Paulsen, J., Ali, T. M. L. & Collas, P. Computational 3D genome modeling using Chrom3D. *Nat. Protoc.* **13**, 1137–1152 (2018).

[42] Love, M. I., Huber, W. & Anders, S. Moderated estimation of fold change and dispersion for RNA-seq data with DESeq2. *Genome Biol.* **15**, 550 (2014).

[43] Tu, S. et al. MAnorm2 for quantitatively comparing groups of ChIP-seq samples. *Genome Res.*

31, 131–145 (2021).
