# Supplementary material for: DiffDomain enables identification of structurally reorganized topologically associating domains
Source: Nat Commun. 2024 Jan 13;15:502. doi: 10.1038/s41467-024-44782-6 (PMC10787792; doi:10.1038/s41467-024-44782-6)
Supplement: Supplementary file 3 — Reporting Summary [file 41467_2024_44782_MOESM3_ESM.pdf]

nature portfolio

Corresponding author(s): Dechao Tian

Last updated by author(s): Nov 30, 2023

Reporting Summary

Nature Portfolio wishes to improve the reproducibility of the work that we publish. This form provides structure for consistency and transparency in reporting. For further information on Nature Portfolio policies, see our Editorial Policies and the Editorial Policy Checklist.

Statistics

For all statistical analyses, confirm that the following items are present in the figure legend, table legend, main text, or Methods section.

n/a	Confirmed	
☐	☒	The exact sample size (*n*) for each experimental group/condition, given as a discrete number and unit of measurement
☐	☒	A statement on whether measurements were taken from distinct samples or whether the same sample was measured repeatedly
☐	☒	The statistical test(s) used AND whether they are one- or two-sided<br>*Only common tests should be described solely by name; describe more complex techniques in the Methods section.*
☒	☐	A description of all covariates tested
☐	☒	A description of any assumptions or corrections, such as tests of normality and adjustment for multiple comparisons
☐	☒	A full description of the statistical parameters including central tendency (e.g. means) or other basic estimates (e.g. regression coefficient) AND variation (e.g. standard deviation) or associated estimates of uncertainty (e.g. confidence intervals)
☐	☒	For null hypothesis testing, the test statistic (e.g. *F*, *t*, *r*) with confidence intervals, effect sizes, degrees of freedom and *P* value noted<br>*Give P values as exact values whenever suitable.*
☒	☐	For Bayesian analysis, information on the choice of priors and Markov chain Monte Carlo settings
☒	☐	For hierarchical and complex designs, identification of the appropriate level for tests and full reporting of outcomes
☒	☐	Estimates of effect sizes (e.g. Cohen's *d*, Pearson's *r*), indicating how they were calculated

Our web collection on statistics for biologists contains articles on many of the points above.

Software and code

Policy information about availability of computer code

Data collection	No software was used for data collection.
Data analysis	All analysis was done on CentOS 7. The source code of DiffDomain (software reported in this manuscript) is publicly available at https://github.com/Tian-Dechao/diffDomain. We used the following open source Python 3.7 packages: hic-straw (1.3.1), pandas (1.3.5), numpy (1.21.6), docopt (0.6.2), matplotlib (3.5.3), statsmodels (0.13.5), h5py (3.8.0), seaborn (0.12.2), and TracyWidom (0.3.0). We compared our method, DiffDomain, with TADsplimer (https://github.com/GuangyWang/TADsplimer, version 1.1), DiffGR (https://github.com/wmalab/DiffGR, commit version 789062e), DiffTAD (https://bitbucket.org/rzaborowski/differential-analysis/src/master/, last updated 2017-02-21), TADCompare (1.8.0), HiCcompare (1.20.0), and HiC-DC+ (1.6.0). Source code of these methods was either downloaded from the latest GitHub repositories or installed by R Bioconductor.

For manuscripts utilizing custom algorithms or software that are central to the research but not yet described in published literature, software must be made available to editors and reviewers. We strongly encourage code deposition in a community repository (e.g. GitHub). See the Nature Portfolio guidelines for submitting code & software for further information.

Data

Policy information about availability of data

All manuscripts must include a data availability statement. This statement should provide the following information, where applicable:

- Accession codes, unique identifiers, or web links for publicly available datasets
- A description of any restrictions on data availability
- For clinical datasets or third party data, please ensure that the statement adheres to our policy

All datasets used in this study are publicly available. Hi-C data of multiple human cell lines and replicates of GM12878 cell line are downloaded from the Gene Expression Omnibus (GEO) database under accession code GSE63525 [https://www.ncbi.nlm.nih.gov/geo/query/acc.cgi?acc=GSE63525] [33]. Hi-C data of patient-derived DIPG, NHA and GBM cell lines and DIPG frozen tissues specimens are downloaded from the GEO database under accession code GSE162976 [https://www.ncbi.nlm.nih.gov/geo/query/acc.cgi?acc=GSE162976]. Hi-C data of mock-infected and SARS-CoV-2 infected A549-ACE2 cells are downloaded from the GEO database under accession code GSE179184 [https://www.ncbi.nlm.nih.gov/geo/query/acc.cgi?acc=GSE179184] [49]. Processed single-cell Dip-C data of multiple cell types in mouse brains are downloaded from the GEO database under accession code GSE162511 [https://www.ncbi.nlm.nih.gov/geo/query/acc.cgi?acc=GSE162511] [52]. The other Hi-C data, DNase-seq data, super-enhancers, and cancer genes are downloaded from the GEO, 4DN, OncoKB, and GeneCards databases, and the data sources are listed in Supplementary Methods A.3.

Research involving human participants, their data, or biological material

Policy information about studies with human participants or human data. See also policy information about sex, gender (identity/presentation), and sexual orientation and race, ethnicity and racism.

Reporting on sex and gender	Sex and gender were not considered in the study design because the study is demonstrating the effective of DiffDomain in analyzing bulk and single-cell Hi-C data, regardless the sex and gender.
Reporting on race, ethnicity, or other socially relevant groupings	Not applicable.
Population characteristics	The only human participants data used in this study is Hi-C of patient-derived DIPG, NHA and GBM cell line and DIPG frozen tissue specimens, which was downloaded from Wang et al. [Ref 1] (GEO: GSE162976).<br><br>Ref 1: Juan Wang, Tina Yi-Ting Huang, Ye Hou, Elizabeth Bartom, Xinyan Lu, Ali Shilatifard, Feng Yue, and Amanda Saratsis. Epigenomic landscape and 3D genome structure in pediatric high-grade glioma. Science Advances, 7(23):eabg4126, 2021.
Recruitment	No participant were recruited by this study. Public data is used (see above) to demonstrate the effectiveness and robustness of DiffDomain in comparing Hi-C data that were obtained from multiple conditions.
Ethics oversight	Not Applicable because all data used in this study is publicly available.

Note that full information on the approval of the study protocol must also be provided in the manuscript.

Field-specific reporting

Please select the one below that is the best fit for your research. If you are not sure, read the appropriate sections before making your selection.

☒ Life sciences ☐ Behavioural & social sciences ☐ Ecological, evolutionary & environmental sciences

For a reference copy of the document with all sections, see nature.com/documents/nr-reporting-summary-flat.pdf

Life sciences study design

All studies must disclose on these points even when the disclosure is negative.

Sample size	No sample-size calculation is performed. More than ten public Hi-C datasets are used, representing Hi-C data with varying sequencing depths, diverse samples, different healthy and disease states, and different species. These dataset are sufficient to demonstrate the effectiveness and robustness of the proposed method.
Data exclusions	Data on ChrY was excluded from the study because it was not available in all bulk and single-cell Hi-C data.
Replication	Replication analysis was performed when possible. Examples include replicating results using different pairs of cell types from different disease states. Additionally, a different numbers of single cells were randomly selected when analyzing single-cell Hi-C data. These replication analysis consistently demonstrate the advantages of DiffDomain over alternative methods and the biological relevance of reorganized TADs.
Randomization	In applying DiffDomain to scHi-C data, individual cells with the same cell type were randomly selected to demonstrate the effectiveness of DiffDomain in analyzing a smaller number of cells. In contrast, when applying DiffDomain to bulk Hi-C data, no randomization is performed. Instead, DiffDomain is applied to bulk Hi-C data from each pair of cell types within the same study. Because DiffDomain is designed to identify reorganized TADs between pairs of conditions, controlling covariates is not relevant in this study.

Blinding	In evaluating the true positive rate and false positive rate of DIffDomain, the labels (reorganized or stable) of TADs were blinded from the model beforehand.

Reporting for specific materials, systems and methods

We require information from authors about some types of materials, experimental systems and methods used in many studies. Here, indicate whether each material, system or method listed is relevant to your study. If you are not sure if a list item applies to your research, read the appropriate section before selecting a response.

Materials & experimental systems

n/a	Involved in the study
☒	☐ Antibodies
☒	☐ Eukaryotic cell lines
☒	☐ Palaeontology and archaeology
☒	☐ Animals and other organisms
☒	☐ Clinical data
☒	☐ Dual use research of concern
☒	☐ Plants

Methods

n/a	Involved in the study
☒	☐ ChIP-seq
☒	☐ Flow cytometry
☒	☐ MRI-based neuroimaging
